# Evidence of defined temporal expression patterns that lead a gram-negative cell out of dormancy

**DOI:** 10.1371/journal.pgen.1008660

**Published:** 2020-03-23

**Authors:** Nandhini Ashok, Carl E. Bauer

**Affiliations:** 1 Department of Biology, Indiana University, Bloomington, Indiana, United States of America; 2 Department of Molecular and Cellular Biochemistry, Indiana University, Bloomington, Indiana, United States of America; Universidad Nacional Autonoma de Mexico Centro de Investigaciones sobre America Latina y el Caribe, MEXICO

## Abstract

Many bacterial species are capable of forming long-lived dormant cells. The best characterized are heat and desiccation resistant spores produced by many Gram-positive species. Less characterized are dormant cysts produced by several Gram-negative species that are somewhat tolerant to increased temperature and very resistant to desiccation. While there is progress in understanding regulatory circuits that control spore germination, there is scarce information on how Gram-negative organisms emerges from dormancy. In this study, we show that *R*. *centenum* cysts germinate by emerging a pair of motile vegetative cells from a thick cyst cell wall coat ~ 6 hrs post induction of germination. Time-lapse transcriptomic analysis reveals that there is a defined temporal pattern of gene expression changes during *R*. *centenum* cyst germination. The first observable changes are increases in expression of genes for protein synthesis, an increase in expression of genes involved in the generation of a membrane potential and the use of this potential for ATP synthesis via ATPase expression. These early events are followed by expression changes that affect the cell wall and membrane composition, followed by expression changes that promote chromosome replication. Midway through germination, expression changes occur that promote the flow of carbon through the TCA cycle to generate reducing power and parallel synthesis of electron transfer components involved in oxidative phosphorylation. Finally, late expression changes promote the synthesis of a photosystem as well as flagellar and chemotaxis components for motility.

## Author summary

Many bacteria are capable of forming long lived dormant cell types that are resistant to harsh environmental conditions. The best characterized example are spores synthesized by several Gram-positive bacteria such as *Bacillus* and *Clostridia* species. Not well characterized are long-lived desiccation resistant cysts synthesized by several Gram-negative bacteria including some pathogens. Several studies have described physiological changes that occur during the germination of Gram-positive spores, however, there are no detailed studies on physiological changes that occur during Gram-negative cyst germination. In this study, we developed a method for isolating cysts from the Gram-negative species *Rhodospirillum centenum* and studied genome wide changes in gene expression that occur as cysts germinate into replicative cells. This study reveals a programed set of expression changes during germination that favors an early generation of a membrane potential for energy production, coupled with expression of gene involved in cellular defense. These early events are followed by large scale physiological changes that promote cellular replication.

## Introduction

Many bacteria enter a period of non-replicative dormancy when faced with nutrient deprivation or harsh environmental conditions. The best studied are dormant long-lived spores by Gram-positive Firmicutes. Many other species are also capable of producing long-lived dormant persister cells that are resistant to antibiotics. Less studied are long-lived dormant cysts synthesized by several Gram-negative species such as *Rhodospirillum centenum*, *Azospirillum* sp., and *Azotobacter* sp. Cysts from these species are not as resistant to environmental stresses as are Gram-positive spores, but they do offer considerable resistance to desiccation and some resistance to heat [1, 2]. Information exists about regulatory events that control the development of Gram-positive spores [3, 4] with just a few studies undertaken on the development of Gram-negative cysts. However, several recent studies do provide at least a baseline understanding of regulatory proteins that control cyst development by *R*. *centenum*, a photosynthetic member of the *Azospirillum* clade [5–11].

Cues initiating Gram-positive spore germination have been well defined and encompass both small molecule nutrient germinants such as amino acids, organic acids, nucleosides and cholesterol, and non-nutrient germinants such as calcium dipicolinate, peptidoglycan fragments, dodecyl amine and even high pressure [12, 13]. Nutrient germinants are thought to bind to a cluster of germination receptors located in the cell membrane [12–15]. This interaction initiates several stages of germination leading to cell wall remodeling, restoration of membrane lipid composition, and core protein movement [12, 13]. Once germination becomes an irreversible process, then there is an outgrowth stage during which there is increased gene expression affecting transport, protein synthesis, and DNA replication [12–14].

In contrast to spore germination, an understanding of Gram-negative cyst germination remains largely unexplored. Several decades ago there were a few microscopic and physiological studies on the germination of cysts from the Gram-negative bacterium *Azotobacter vinelandii*. These microscopic studies observed that *A*. *vinelandii* cysts underwent division during the germination process just before rupture of a thick exine exopolysaccharide coat that encloses individual cysts [16, 17]. Additional physiological analyses also showed that *A*. *vinelandii* cysts exhibit rapid induction of protein synthesis, nucleic acid synthesis, and then respiration upon induction of germination [18]. These early events were followed by changes in metabolism, initiation of DNA synthesis, and nitrogen fixation just before emergence from the exine coat [18]. Regulatory circuits that control the induction of these ordered processes in germinating *A*. *vinelandii* cysts are not yet defined.

In this study, we have analyzed the germination of *R*. *centenum* cysts using a combination of time-lapse microscopy coupled with RNA-seq based transcriptomic analysis. Results from time-lapse microscopy show that the germination period is ~ 6 hrs in length terminating with cyst wall hydrolysis and the emergence of more than one motile vegetative cell. Transcriptomics performed at defined periods in the germination developmental pathway show that genes responsible for protein synthesis, energy production and cell defense are among the first to be expressed. This ramp-up in energy production was followed by periods of DNA replication, flagellar synthesis, and cell wall remodeling. The combination of time-course microscopy, coupled with transcriptomic data, provides the first reported molecular chronology of events that take place during the emergence of a Gram-negative cyst from dormancy.

## Results and discussion

### Microscopic analysis of cyst germination

**Fig 1:**
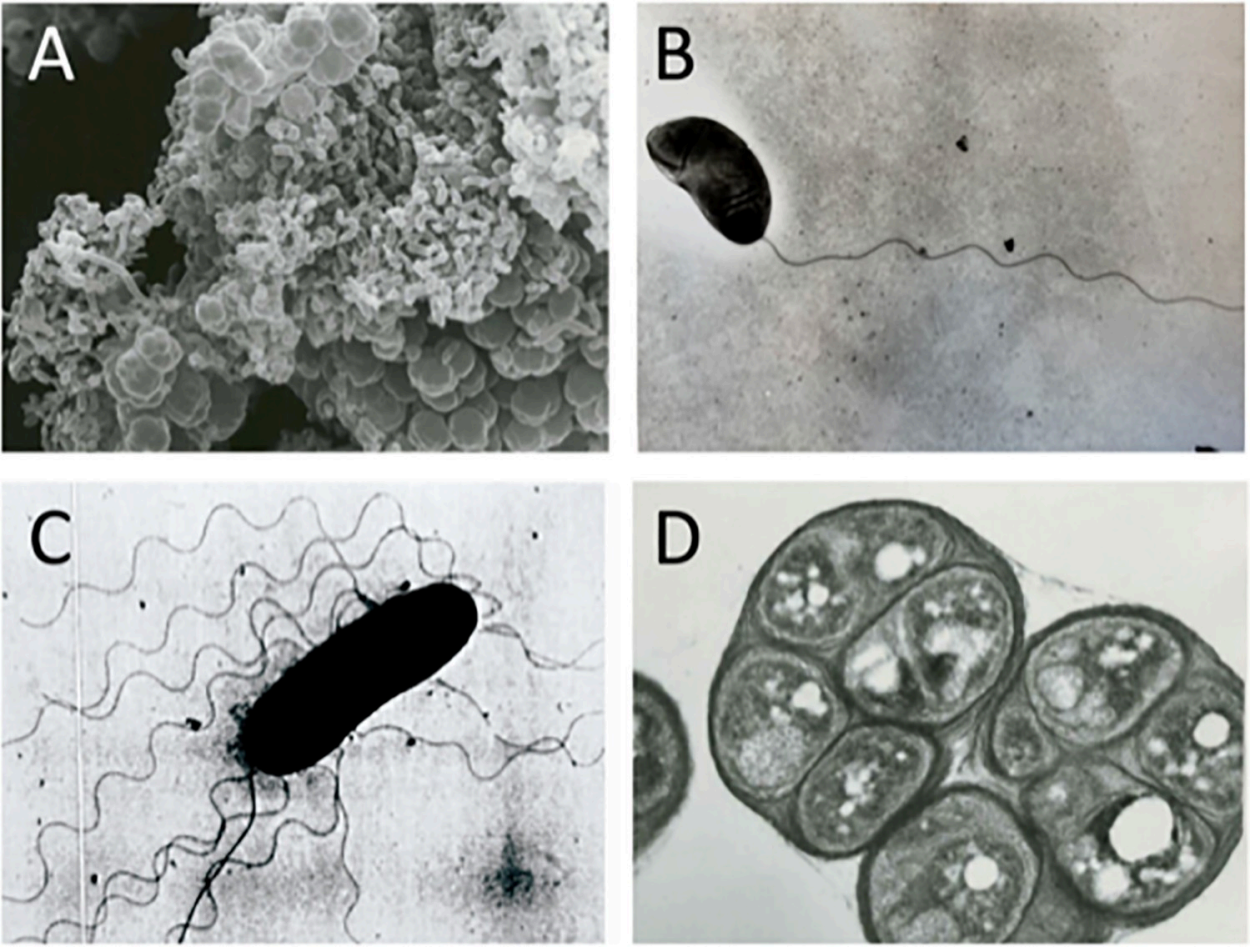
SEM and TEM electron micrographs of *R*. *centenum*. A) SEM analysis of an *R*. *centenum* colony undergoing transition from vegetative (vibrioid/rod cells) to cyst forms in CENSPY medium as described in ref [1]. TEM image of *R*. *centenum* swim cells in liquid medium containing a single sheathed polar flagellum. C) TEM of swarm cells grown on agar solidified medium with a sheathed polar flagellum and numerous lateral flagella. D) TEM of cyst cells that form tightly packed clusters of cells coated with a thick exine polysaccharide layer.

Fig 1A shows a scanning electron micrograph of an *R*. *centenum* colony undergoing a transition from vegetative to cyst phases. The individual viroid shaped replicative cells are quite distinct from cysts that are large clusters of dormant cells encased in a thick exine layer of exopolysaccharides. Transmission electron micrographs of replicative and cyst cells also highlight substantial morphological differences between these two cell types (Fig 1B–1D). Liquid grown *R*. *centenum* vegetative cells are viroid in shape with a single sheathed polar flagellum (Fig 1B), while vegetative cells grown on an agar solidified media exhibit both a sheathed polar flagellum and numerous unsheathed lateral flagella (Fig 1C). In contrast, dormant cysts are round non-flagellated cells encased within a thick exine polysaccharide extracellular wall (Fig 1D). Cyst cells also are replete with large pools of highly refractive polyhydroxy butyric acid (PHB) that is thought to be used as an internal energy source for long-term cyst survival and emergence from dormancy [19].

**Fig 2:**
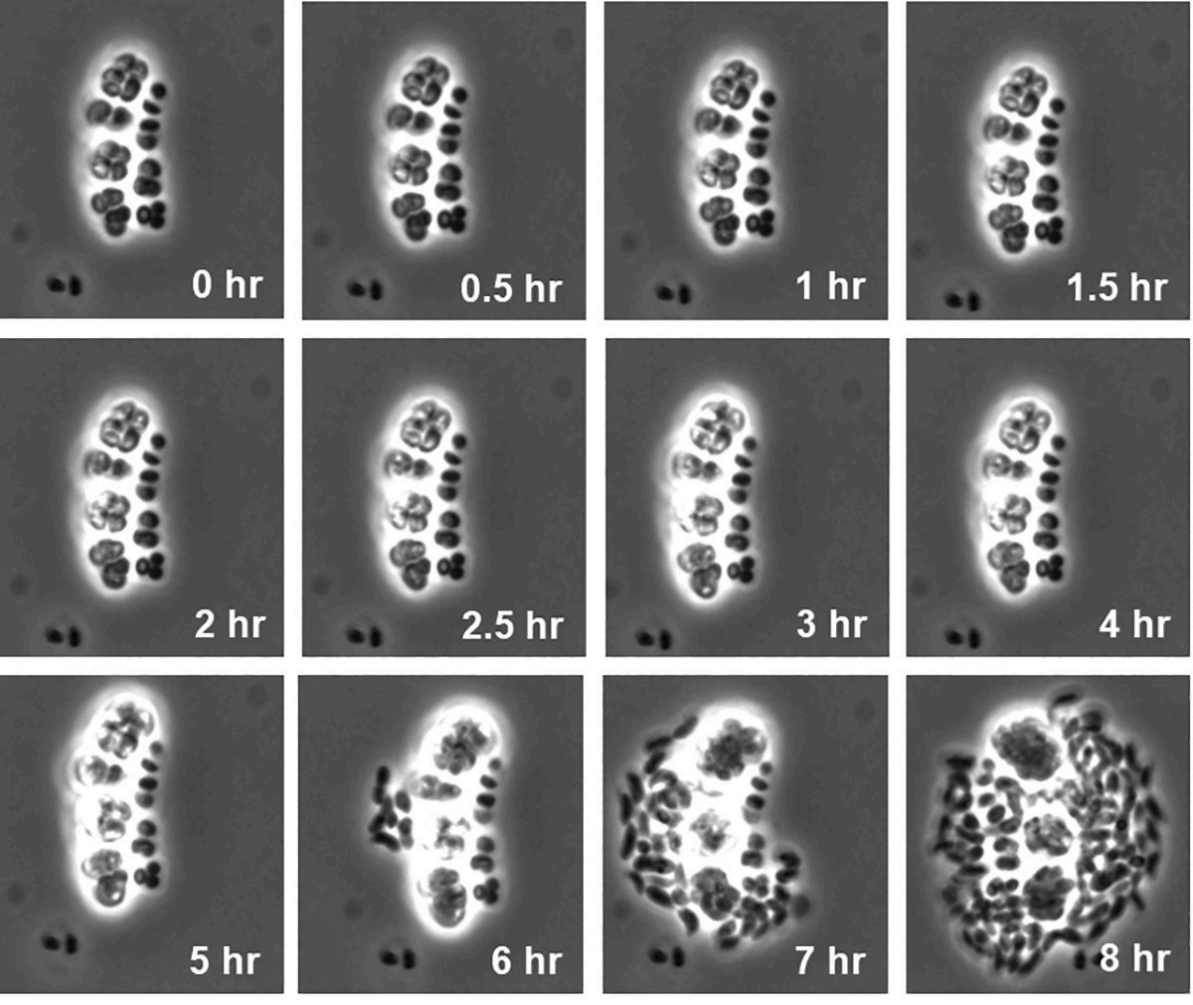
Oil immersion microscopic analysis of refractile cyst cells undergoing germination over an 8-hour period.

The process of cyst germination was monitored by undertaking time-lapse live cell imaging of cysts placed onto nutrient rich CENS agar (Fig 2 and S1 Movie). During early phases of the germination process, the cyst cluster undergoes a slight swelling in size reminiscent of swelling of a *Bacillus* spore that occurs while they take up water from the environment [12, 20]. There is also a distinct movement of phase bright cells within the exine polysaccharide extracellular wall ~30 minutes of the initiation of germination. The first emergence of vegetative cells from the exine layer typically occurs 5.5 hrs post induction of germination with most cysts germinated by 6 hrs. Typically, pairs of cells emerge together from cysts indicating that cells divide before hydrolysis of the exine polysaccharide extracellular wall. Finally, after emergence there is an empty exine polysaccharide extracellular wall left behind that can be visualized as dark empty “husks” as the germinating vegetative cells continue to divide outside of the exine wall.

### Overview of cyst germination transcriptomic analysis

We undertook RNA-seq based transcriptomic analysis at various time points during cyst germination to assess temporal changes in expression that occur when cells leave dormancy and enter a period of replication. We first developed a method to remove any remaining vegetative cells from cyst inducing growth medium and then measured the amount of cyst enrichment by flow cytometry. Cysts used in this study were typically >95% enriched (S1 Fig).

Using isolated cysts, a pilot experiment was initially undertaken to address how quickly changes in expression are observed post initiation of germination. After assessing these results, we undertook replicate studies (three biological replicates) where germination was induced by transferring isolated cyst into nutrient-rich CENS medium from which aliquots were removed for RNA extraction. RNA samples were taken every ½ hour for the first 3 hours, followed by every 1 hour until reaching 8 hrs. Microscopic analysis shows that by 8 hours, the cysts were fully germinated so this last time point also provided a reference of RNA levels observed from vegetative cells relative to the RNA levels observed from fully dormant cysts. RNA extracted from every time point (0.5, 1, 1.5, 2, 2.5, 3, 4, 5, 6, 7, and 8 hr) was compared to RNA extracted from dormant cysts (0th hour) with regions considered differentially expressed (DEG) if the log_2_ value from this comparison was greater than or equal to ±1.5 with a p-value <0.05. Finally, RNA-seq data sets were independently validated by assaying expression changes of five genes at four time points (0, 0.5, 2.5 and 6 hr) using quantitative PCR (qRT-PCR). For each gene and time point, log_2_fold change with respect to the 0^th^ hour from qRT-PCR was calculated and plotted against the same fold changed obtained from independent RNA-seq data sets (S2 Fig). The obtained *R*^*2*^ value between qRT-PCR and RNA-seq data sets for these genes shows excellent correlation with an *R*^*2*^ = 0.91.

During the time course of induction, we observed that 40.64% of the *R*. *centenum* genome (1,667 genes) underwent significant changes in expression relative to fully dormant cysts (S1 Table). Given this large number of gene expression changes, we analyzed the data by first binning into clusters of orthologous groups (COGS) where genes that control similar processes were individually analyzed (S3 Fig). In the sections below, we discuss individual COGS that best highlight molecular events that lead to the emergence of a fully functional vegetative cell. Finally, we compared transcriptomic data collected during germination in this study to a prior study from our laboratory that analyzed transcriptomic changes in expression that occur during cyst formation [21]. Interestingly, 317 genes exhibit DEG in both data sets with 194 of these showing opposing regulation defined as upregulation during cyst formation and downregulation during cyst germination or vice versa (S4 Fig). Comparison of gene expression changes during germination with that observed during cyst formation contributed to a more in-depth insight into both the germination and encystation processes in this Gram-negative species.

### Early events initiating 0.5 to 1.5 hrs

#### Translation machinery

**Fig 3:**
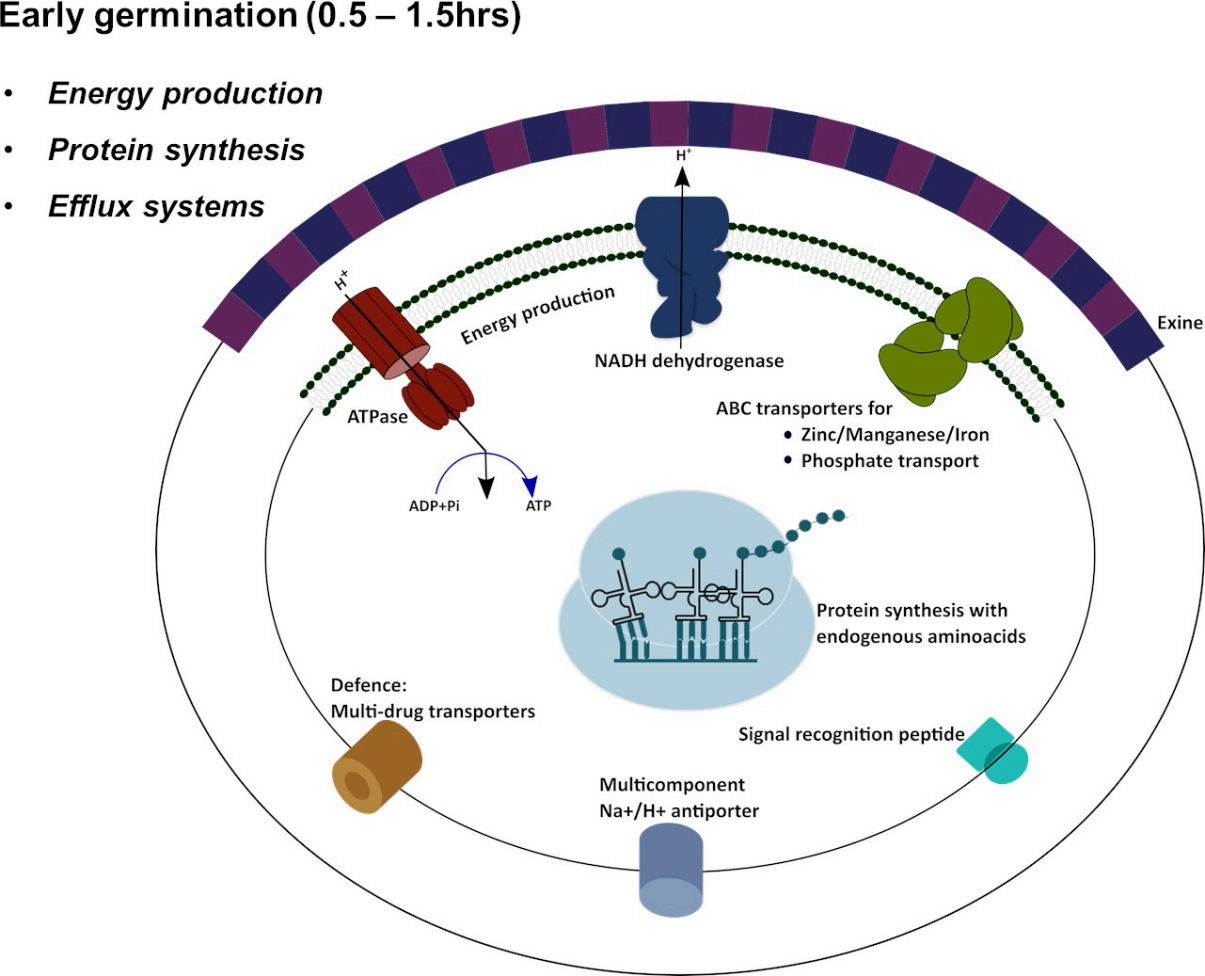
Diagram of cellular processes that undergo an increase in expression during the early germination period (0.5 to 1.5 hrs.).

An important set of genes expressed earliest in germination include those that encoded proteins of the large and small ribosomal subunits (Fig 3 and S5A and S5B Fig). This group of genes uniformly showed significant increases in expression at the earliest 0.5 hr time point and continued to increase in expression throughout germination. Most initiation factors (IF-1 and IF-2), elongation factors (Tu, G and P) and release factors involved in translation also show significant upregulation early in germination with sustained increases in expression throughout (S5C Fig). The one exception is the elongation factor G (EF-G) coded by RC1_1873 that is inexplicably downregulated during germination with the caveat that the *R*. *centenum* genome also codes for two additional EF-G's (RC1_0708 and RC1_3537) that both exhibit early increases in expression. Note that early induction of protein synthesis is known to occur soon after the initiation of *Bacillus* spore germination [22].

In addition to synthesis of translation machinery, genes coding for proteins involved in protein folding, such as HSP40, and HSP90 and GroEL, are also upregulated during this early time point with some showing significant regulation across the time spectrum (early to middle–SurA^RC1_1563^, CcmG^RC1_3021^, middle–HSP20, HSP10 and middle to late–RC1_0986, RC1_2666) (S5D Fig). There is also upregulation of *ftsY*^*RC1_2195*^ –a gene involved in the signal recognition particle system for proper insertion of membrane proteins (Fig 3 and S5E Fig) [23, 24].

#### Charging the membrane for ATP production

Another set of genes expressed at nearly the same early time as those involved in protein synthesis are those involved in generating a membrane potential. Specifically, at 1.5hr after induction of germination there is rapid induction of components of NADH dehydrogenase–complex 1 that moves electron and protons from NADH to the ubiquinone pool (Fig 3, S6A Fig). Complex 1 also shuttles 4H+ across the cytoplasmic membrane so its expression helps germinating cysts develop a membrane potential that can be used for energy production (Fig 3). Concurrently, there is early (0.5 to 1 hr) induction of genes coding for subunits of an F-type H^+^-transporting ATPase (complex V) that utilizes a proton gradient to generate ATP (Fig 3 and S6B Fig). Starting at 0.5 hr, and peaking at ~2.5hrs, there is also induction of genes coding for components of pyruvate dehydrogenase that utilizes pyruvate to generate NADH, acetyl CoA and CO_2_ (S6C Fig). NADH from this reaction could also be used by NADH dehydrogenase to sustain energy production via ATPase. We also note a 4 to 5-fold in upregulation of pyruvate dehydrogenase genes during cyst formation, just like that observed during cyst germination [21] (S6C Fig).

#### Drug resistance

Another set of genes that undergo early expression increases are three genes coding for components of two multidrug exporters (*emrA*^RC1_4050^, *emrB*^RC1_4051^, and *acrB*^RC1_2565^) (Fig 3, S7 Fig). The macrolide transporter MacBRC1_2439 also gets upregulated, though slightly later at 2 hrs. This expression pattern presumably confers resistance to extracellular toxins, thereby providing germinating cysts a defense mechanism during this transient and vulnerable stage. Expression of multidrug transporters during germination is also seen in *Bacillus subtilis* [25]. Note that the expression of these three resistance genes are markedly reduced when cells undergo the development of cysts. Since cysts are metabolically dormant, there is presumably little need for expression of these drug exporters while in the cyst phase.

Overall, these results collectively show that germinating cysts initially induce expression of genes involved in protein synthesis and NADH dehydrogenase that utilizes NADH for generating a membrane potential. Concurrently, there is the expression of ATPase that uses a membrane potential for the production of ATP. These results indicate that there is an initial preference for both the production of protein synthesis machinery and the initiation of energy production. Finally, there is also an early expression of proteins involved in defense against antimicrobial compounds.

### Early to middle events (1–3 hrs.)

#### Cell wall and membrane remodeling

**Fig 4:**
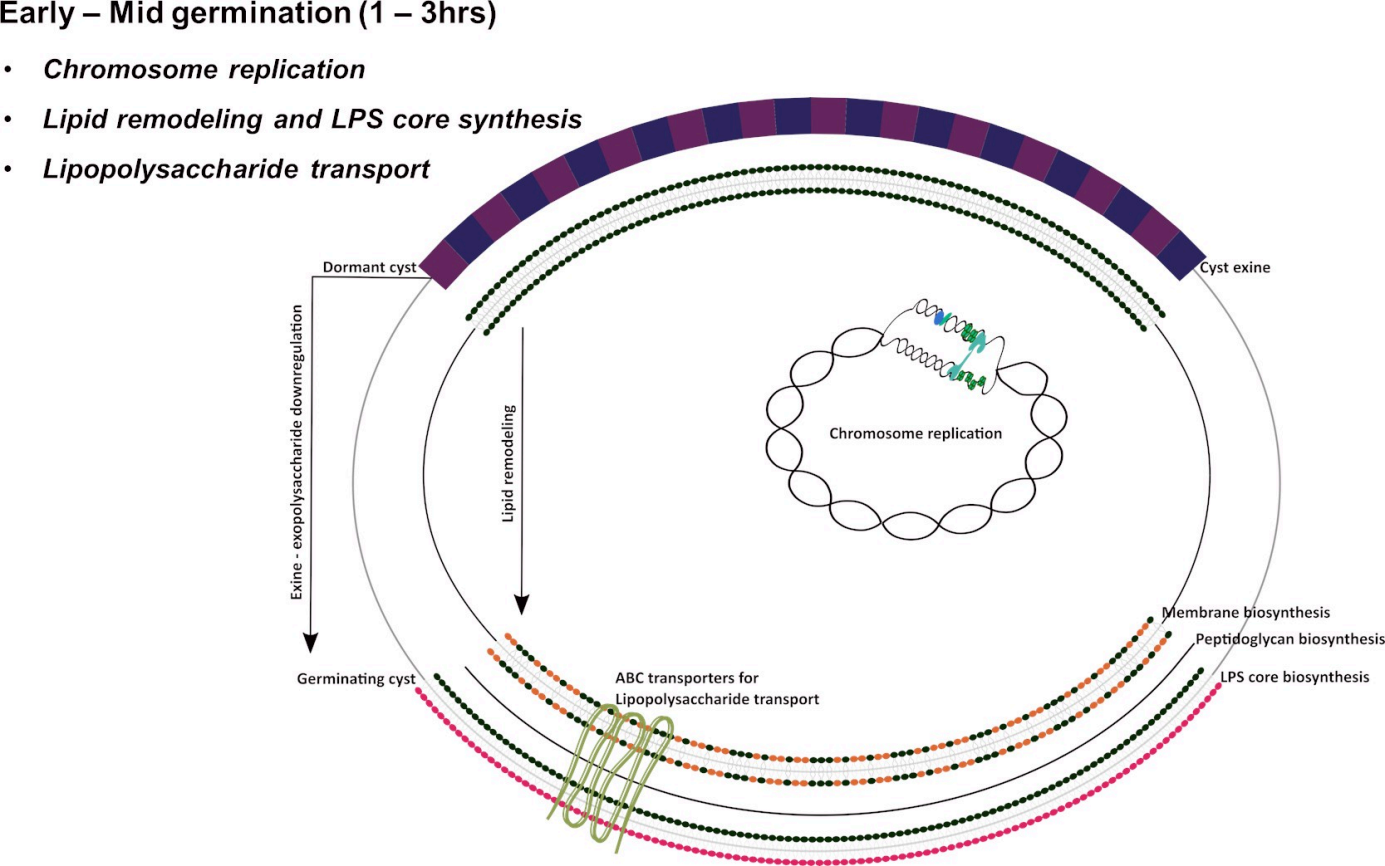
Diagram of cellular processes that undergo changes during the early to middle germination period (1 to 3 hrs.).

The cell membrane and outer cell wall are quite different between desiccation resistant cyst cells that do not undergo replication versus replicating vegetative cells (Fig 4). In *A*. *vinelandii*, where the cysts cell membrane has been best studied, phospholipids present in vegetative cells are converted to 5-*n*-alkylresorcinols and 6-*n*-alkylpyrones during cyst development [26, 27]. These alkyl cyst lipids are thought to create a more impermeable membrane matrix, thereby conferring desiccation resistance [26, 27]. Not surprisingly, the *fabZ*, *fabF*, *fabH*, *fabG*, and *fabK* genes involved in fatty acid synthesis [28] all exhibit upregulation in the early to mid-timepoint (S8A Fig).

Cysts are also coated with a very thick layer of exopolysaccharides, so it is also not surprising that there are many changes in expression of genes involved cell wall metabolism early in germination (starting at ~1 hr) and throughout the germination period (Fig 4, S8 Fig, S9 Fig). For example, genes involved in peptidoglycan biosynthesis (*murb*, *murG*, *murD*, *mraY*, *murF*, *ddiB* and *uppS*) (S8B Fig) [29] and genes involved in building components of the outer membrane, such as those involved in synthesis of the lipid A-core (*lpxA*, *hrtB*, *RC1_0231*, *RC1_1918*) (S9A Fig) show upregulation in early to mid-timepoints. It is to be noted that the same *mur* peptidoglycan biosynthesis genes decrease expression during development of cysts (S8B Fig). However, genes involved in synthesis of the core oligosaccharide layer and/or O antigen layer (*algC*, *galU*, *rkpK*, *rfbA*, *rfbC*, *rfbD*) all exhibited upregulation later at ~4–6 hours after induction of germination (S9B Fig) indicating later completion of cell wall synthesis around just before the cyst husk breaks open. Interestingly, genes involved in anhydromuropeptide recycling (*nagA* and *RC1_0623*), a process that happens during cell growth [30], shows downregulation (S9C Fig). This result suggests an absence of cell wall recycling during the transition of cysts from dormancy into vegetative cells.

A set cell wall of genes coding for enzymes involved in the production of UDP-α-D-galactose (*galE* and *exoB*) and GDP-L-fucose (*RC1_3992*) undergo opposing expression patterns (S9D Fig). Specifically, these genes exhibit increased expression during cyst development and decreased expression during cyst germination. This expression pattern suggests that these genes may code for enzymes involved in modulating cell wall changes specific to the cyst cell wall.

#### DNA replication and Nucleotide metabolism

Time-lapse videos of germination (see Fig 2 and S1 Movie) indicate that motile cells emerge from the exine coat in pairs suggesting that cellular replication occurs as part of the germination process. This hypothesis is supported by the expression pattern of genes associated with DNA replication. For example, 2.5 hours after induction of germination there is significant upregulation of two genes coding for subunits of DNA polymerase III (*RC1_0787*, *RC1_2553*) as well as *ruvB* coding for a DNA replication helicase (Fig 4, S10A Fig). At 2–2.5 hrs. post initiation of germination, there is maximal expression of the chromosome partitioning protein (*parB*^*RC1_2813*^) [31]. We suspect that dormant cyst cells do not contain multiple replication forks and note that it takes 60 to 90 min to replicate a 4 Mbp prokaryotic genome. Consequently, the initiation of DNA replication would likely occurs well before the completion of chromosome replication and subsequent segregation into daughter cells that occurs late in germination [32, 33]. Along these lines there is also ~4-fold increased expression of *hupB* that codes for HU that is involved in chromosome packing at 2.5 hrs post initiation of germination (S10A Fig). Finally, most of these DNA replication genes are also down-regulated during cyst development which is not surprising as dormant cysts are not undergoing chromosome replication.

In regard to nucleotide metabolism, there is upregulation of a transcriptional repressor of ribonucleotide reductases (*nrdR*^*RC1_1341*^) concurrent with downregulation of ribonucleotide reductases (*nrdR*^*RC1_1341*^) that are involved in the synthesis of deoxyribonucleotides (S10B Fig). While down-regulation of ribonucleotide reductase may seem contradictory to the hypothesis that DNA replication occurs during early/mid germination, Gram-positive spores are known to contain a significant reserve pool of deoxyribonucleotides [34]. Consequently, it is also possible that Gram-negative cysts may likewise retain a deoxyribonucleotide reserve pool. Along this vein, we note that at ~1hr, and proceeding throughout germination, there is a significant increase in expression of a gene coding for a nucleoside-diphosphate kinase (*RC1_1554*) (S10C Fig). This enzyme promotes an exchange of phosphate between different nucleoside triphosphates that would presumably help promote a balanced equilibrium between the various nucleoside triphosphate pools [35].

Finally, we also observed early/mid upregulation of *RC1_1499* involved in the synthesis of (p)ppGpp (S10C Fig). This expression pattern during germination is opposite to that observed during cyst development. Furthermore, spores of *Bacillus megaterium* are known to synthesize (p)ppGpp minutes after the induction of germination [36]. In addition, there is mid to mid/late expression of an inorganic pyrophosphatase gene *ppa* (S10D Fig). Pyrophosphate is generated as a byproduct of DNA and RNA synthesis and needs to be broken down to inorganic phosphate to maintain a balanced phosphate pool, which is essential for cellular viability [37, 38].

### Middle to late events (3–6 hrs.) and later (6–8 hrs.)

#### Central metabolism

**Fig 5:**
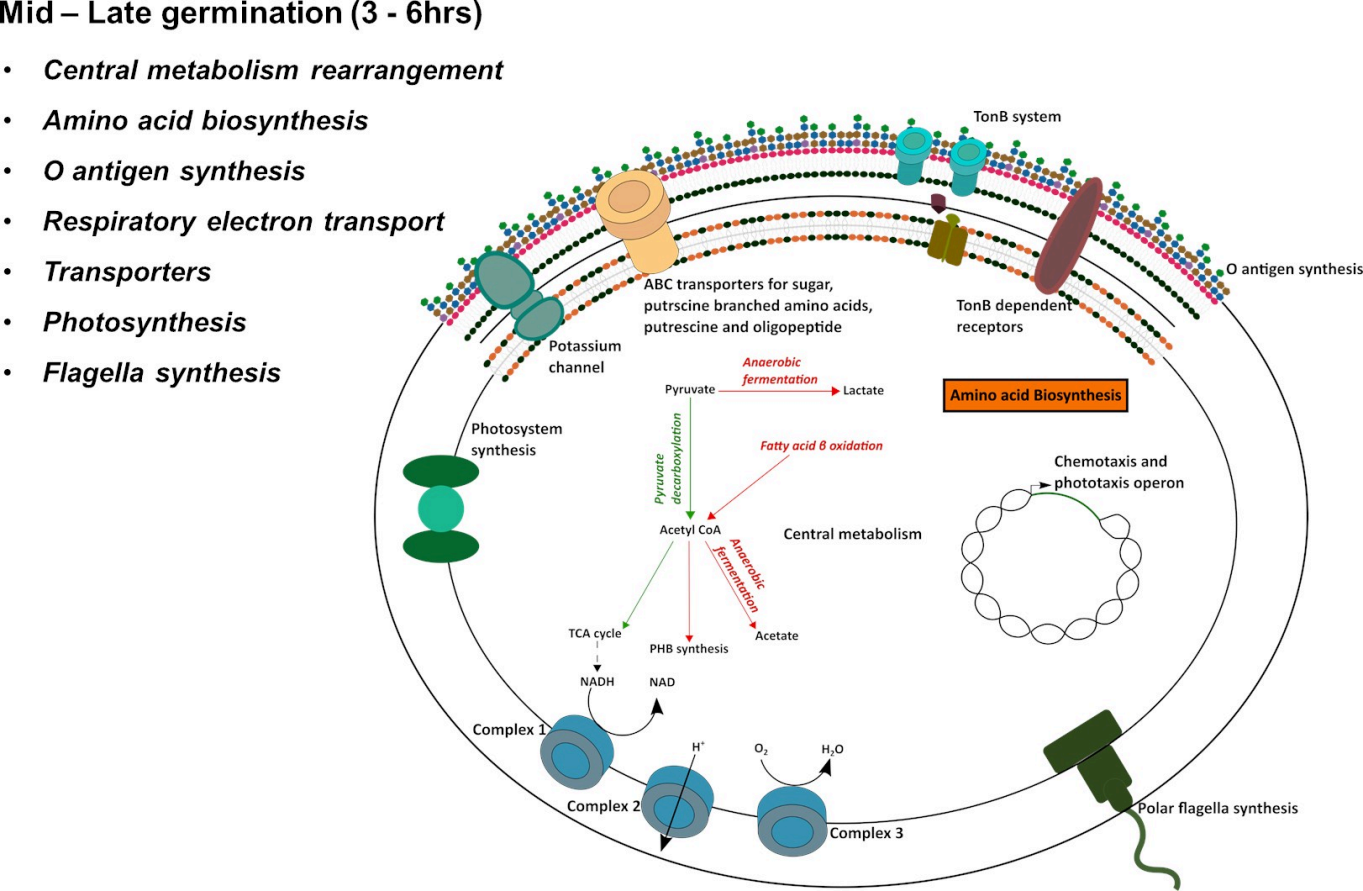
Diagram of cellular processes that undergo changes during the middle to late germination period (3 to 6 hrs.).

There is a clear increase in expression of genes coding for enzymes involved in central metabolism in the mid to late phase of the germination cycle. Specifically, there are significant increases in the expression of genes coding for enzymes that flow carbon from pyruvate into the TCA cycle which drives NADH production (Fig 5, S6C Fig and S11A Fig). There is also increased expression of respiratory components such as succinate dehydrogenase (Complex II), cytochrome *bc*_*1*_ (Complex III), as well as components of a *cbb*_*3*_ type cytochrome *c* oxidase (Complex IV) (S11B–S11D Fig) that utilizes electrons from NADH and succinate dehydrogenase to drive oxidative phosphorylation. There are also mid/late increases in expression of *ubiB* that codes for a protein catalyzing the first monooxygenase step in ubiquinone biosynthesis. This is a step in ubiquinone biosynthesis that is proposed to have a regulatory role in the synthesis of ubiquinone (S11E Fig) [39].

**Fig 6:**
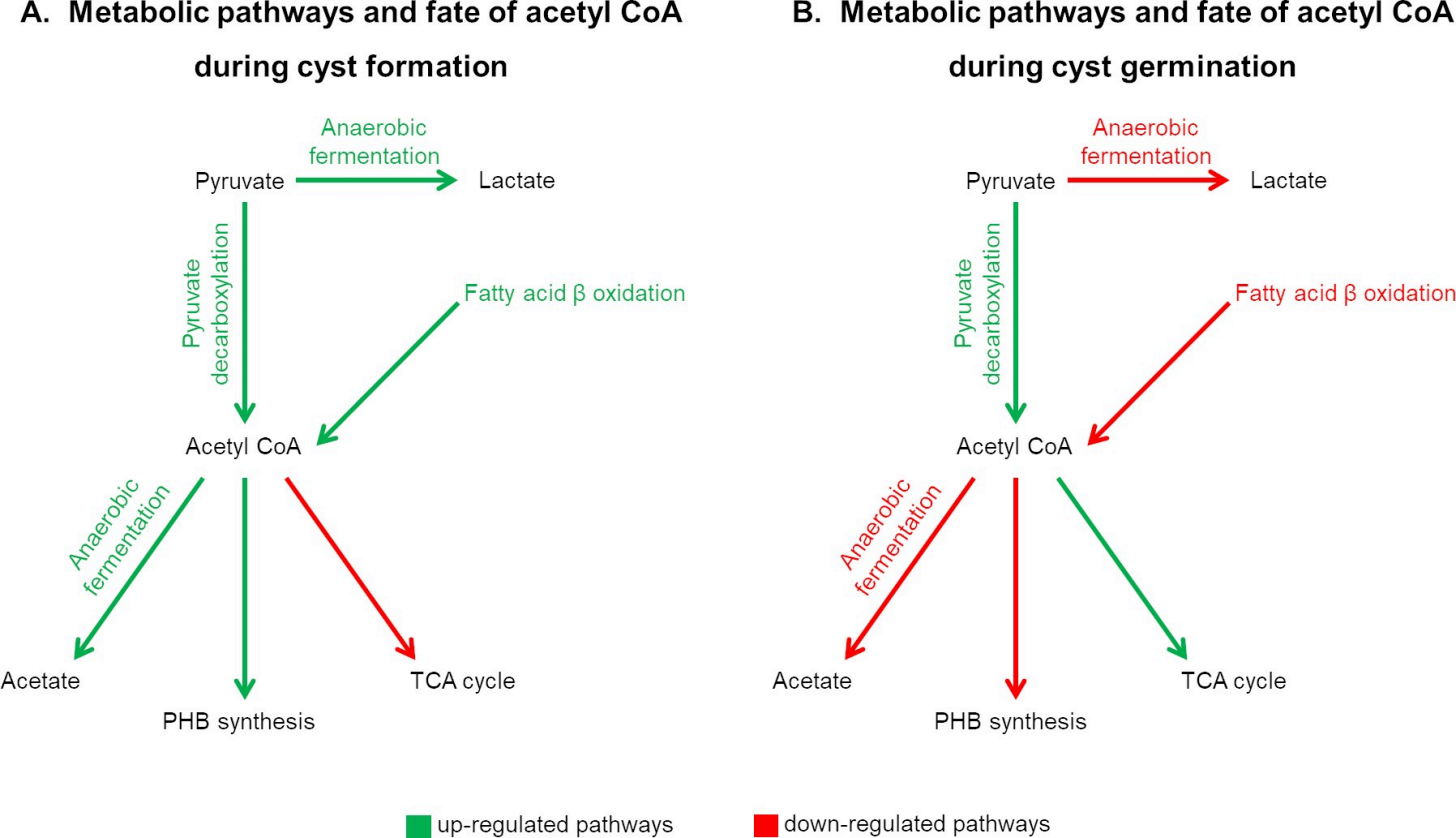
Summary of gene expression changes that affect central metabolism. A) In cells undergoing the formation of cysts, there is increased expression (green arrows) of genes involved in anaerobic fermentation and production of polyhydroxybutyrate (PHB). Concurrently, there is also reduced expression (red arrows) of genes involved in aerobic respiration via the TCA cycle (expression data from [21]). B) In germinating cyst cells there is increased expression of genes promoting aerobic energy production via the TCA cycle as well as a reduction in anaerobic fermentation and PHB synthesis.

At the same time that germinating cysts are ramping up expression of genes involved in aerobic respiration, they are also significantly reducing expression of genes involved in anaerobic fermentation that lead to the production of acetate and lactate respectively (Fig 5 and S12A and S12B Fig). The same reduced expression also occurs for genes that code for enzymes involved in fatty acid β-oxidation that promotes the brake-down of fatty acids into acetyl CoA, genes for the conversion of acetyl CoA to acetate, and genes that code for enzymes involved in PHB synthesis (S12C and S12D Fig). Interestingly, all observed changes in gene expression that affect central metabolism during germination are inverse to what has been observed to occur during cyst development [20] (Fig 6). This opposing regulation fits the logical narrative that cyst development and cyst germination are bifurcating processes and also validates both data sets. Furthermore, it is rather clear from these observed changes that cysts derive energy via anaerobic fermentation while germinating cells reprogram metabolism to derive energy via oxidative phosphorylation.

#### Amino acid biosynthesis

While the protein synthesis machinery showed upregulation at the earliest time point, amino acid biosynthetic pathways and many aminoacyl tRNA synthetases exhibit upregulation only at the middle to late time points (S13 Fig). We suspect that endogenous amino acids pools are present in dormant cysts for use during initial protein synthesis, as seen in germinating spores of *Bacillus megaterium* [40]. We speculate that protein synthesis with newly synthesized amino acids happens only after the middle time point.

While most genes involved in amino acid biosynthesis are upregulated in this data set, there are a few interesting examples of decreased expression of important genes in this category. This includes reduced expression of two NifS isomers (RC1_0478 and RC1_1616) coding for putative cysteine desulfurases that generates Ala and elemental sulfur from Cys (S13 Fig). This reaction has been implicated in the generation of sulfur for use in the assembly of iron-sulfur centers in nitrogenase as well as for the sulfur in the ring of biotin [41, 42]. Germination was initiated in a nitrogen-rich complex medium; so, these germinating cells may be nitrogen replete and therefore not assembling an active nitrogenase. This hypothesis is supported by reduced expression of the small chain of glutamate synthase that is also needed for nitrogen assimilation (S13 Fig).

#### Photosynthesis

At ~3 hours post induction, when cells are ramping up expression of genes involved in TCA cycle/oxidative phosphorylation, these cells are also ramping up expression of genes involved in the synthesis of a photosystem (Fig 5 and S14 Fig). Specifically, several *bch* and *hem* genes coding for enzymes for bacteriochlorophyll and heme biosynthesis exhibit elevated expression 3 hrs. post induction of germination. At ~6 hrs. post induction of germination, the *puf* and *puh* genes coding for light harvesting I and reaction center structural components also ramp up expression (S14 Fig). This expression pattern presumably ensures that mature vegetative cells exit dormancy fully capable of producing energy via photosynthesis.

#### Motility

*R*. *centenum* is known to synthesize two different flagella types, a sheathed polar flagellum and unsheathed lateral flagellum [43, 44]. Two sets of genes are present in the chromosome that are dedicated either to lateral or polar flagella components with phylogenetic analysis indicating that lateral flagellar genes were obtained via lateral gene transfer [45, 46]. In liquid medium, these cells only synthesize a polar flagellum, whereas when grown on an agar surface they synthesize both flagellum types [44, 47]. Cysts in this study were obtained from agar grown cultures that would synthesize both flagellar types while cyst germination occurred in liquid medium where only the polar flagellum is synthesized. It is therefore not surprising that we observed a ramp-up of polar flagellar gene expression and down-regulation of lateral gene expression during germination (S15A and S15C Fig, respectively).

In addition to flagellar structural genes, the genome of *R*. *centenum* also codes for three independent Che-like gene clusters [45]. The Che1 gene cluster involved in regulating chemotaxis and phototaxis [48, 49], the Che2 gene cluster involved in regulating lateral flagellum synthesis [44], and the Che3 gene cluster involved in regulating cyst cell development (discussed in the Signal Transduction section below) [8, 10, 50]. Interestingly the Che1 and Che2 gene clusters both mimic their roles relative to polar and lateral flagella gene expression. Specifically, the *che1* operon responsible for phototactic and chemotactic behavior, shows a significant increase in expression ~ 3rd hours onwards as do the expression of most polar flagellar structural genes (S15A and S15B Fig). Likewise, the *che2* operon that is involved in the control of lateral flagellar synthesis [44] is downregulated during germination as are that of the lateral flagella structural genes (S15C and S15D Fig).

#### R body genes

During middle/late germination, there is also a notable ramp-down in the expression of genes that code for R bodies (*RC1_ 1995*, *RC1_ 1997*, *RC1_ 1999*) (S16 Fig). R bodies are a proteinaceous coiled ribbon that is thought to confer resistance to predation [51]. During cyst development, R body gene expression is significantly ramped-up presumably in response to the fact that cysts are non-motile, so the production of a predation defense is likely advantageous.

### Gene expression events that span early through late germination

#### Transport

While it is likely that transport of organic and inorganic materials across the cell wall/membrane is limiting during dormancy, transport systems most likely become important during cyst germination. For example, spores of *Bacillus* exhibit high specificity of timing on what can enter and exit the cell at different periods of germination and outgrowth [12, 52]. A varied expression pattern for genes involved in transport during *R*. *centenum* cyst germination likewise shows the existence of similar selective permeability. For example, genes coding for zinc/manganese/iron transport system (*troC*^*RC1_2461*^ and *troD*^*RC1_2462*^) show upregulation at early time points (0.5 hrs.– 1.5 hrs) similar to that observed in *Bacillus* spore germination (Fig 3 and S17A Fig) [25]. However, unlike *Bacillus*, genes coding for ABC transporters of phosphate (*pstS*^*RC1_3262*^, *pstC*^*RC1_3263*^, and *pstB*^*RC1_3265*^) also show early upregulation (Fig 3 and S17B Fig). Though single genes involved in the efflux of monovalent cations show both early (*RC1_2885*) and late (*RC1_3663*) downregulation (S17C Fig), genes coding for a multicomponent Na+/H+ antiporter (*RC1_3665*, *RC1_3666*, *RC1_3667*, *RC1_3668* and *RC1_3669*) [53, 54] show early upregulation (Fig 3 and S17C Fig) indicating an efflux of monovalent cations early in germination, like that observed during *Bacillus* germination [12, 25]. Also, it is of relevance here to note that a gene for K+ stimulated pyrophosphate energized sodium pump goes up late (between 6–8 hours) in germination (S17D Fig). This gene is known to code for a pump that pushes sodium ions across the cytoplasmic membrane by using energy from the hydrolysis of pyrophosphate [55]

Later during germination at ~2 hrs, there is increased expression of lipopolysaccharide transporters (*RC1_0820*, *YjgP*^*RC1_0756*^, *YjgP*^*RC1_0757*^, *and YjgP*^*RC1_1564*^) which corresponds to the time of increased expression of enzymes involved in LPS synthesis (Fig 4, S18A Fig and S9A Fig). Aptly, *RC1_0820*, and *RC1_1564* were previously shown to be downregulated during the formation of non-dividing dormant cysts (S18A Fig) [21]. Also, genes annotated to be involved in molybdenum transport (RC1_2216, RC1_2218, RC1_2219) show downregulation during this time point (S18B Fig).

ABC transporters specific for sugar transport (*RC1_0249*, *RC1_0250*, and *RC1_0251*) show upregulation at the middle to late timepoint ~4 hrs into germination at the same time that enzymes for central metabolism (notably component of the TCA cycle) are ramped up in expression. We speculate the necessity to rapidly supply a germinating cyst with sugar for efficient energy generation. (Fig 5 and S19A Fig) [21].

Genes involved in branched-chain amino acid transport (*livG*^*RC1_1833*^), putrescine transport (*potI*^*RC1_1457*^, *potH*^*RC1_1458*^, and *potG*^*RC1_1459*^), and oligopeptide transport (*oppB*^*RC1_3364*^ and *oppC*^*RC1_3365*^) are upregulated during the early, middle and late time points, respectively (Fig 5 and S19B, S19C and S19D Fig). A gene for potassium transport (*RC1_0384*) is upregulated late in germination (6 hours) (Fig 5 and S19E Fig), unlike *Bacillus* where potassium transport is known to be upregulated early in spore germination and outgrowth [25]. Conversely, genes in the operon involved in the active transport of potassium (*kdpC*^*RC1_0434*^ *and kdpA*^*RC1_0436*^) are all downregulated (S19F Fig). Also, unlike *Bacillus*, where copper transport is upregulated [25], this data set shows down-regulation of the copper transport gene (*nosF*^*RC1_3910*^) late in germination (S19G Fig).

It is at the middle to late timepoints where genes of the *tonB* transport system (*exbB*^*RC1_0681*^, *exbD*^*RC1_0682*^, *and exbD1*^*RC1_0683*^) that are involved in the transport of iron, nickel, small molecules, vitamin B_12_ and carbohydrates show upregulation (Fig 5 and S20A Fig). Eleven TonB dependent receptors also show differential regulation during various times. Specifically, 6 TonB receptors are downregulated (*RC1_0278*, *RC1_2392*, *RC1_2946*, *RC1_3723*, *RC1_3920* and *RC1_3980*) while 5 are upregulated (*RC1_0402*, *RC1_0463*, *RC1_0580*, *RC1_0945* and *RC1_3711*) including *RC1_0945* which is a siderophore specific TonB receptor (Fig 5 and S20B Fig). Interestingly, many of these TonB system genes also show an opposing expression pattern during cyst formation (S20A and S20B Fig) [21].

#### Transcription and signal transduction

To access whether it is advantageous to initiate germination, dormant cysts presumably monitor both their extracellular environment and their internal metabolic state. Once it is perceived that its advantageous to germinate then these cells initiate an ordered cascade of gene expression changes that lead to germination. Not surprisingly, a large number of genes coding for sigma factors, other transcription factors, and signaling proteins change expression throughout the germination cycle. Presumably some function as master regulators and others as downstream players that orchestrate the germination event.

Early on at ~1.5 hrs, there is ramped up expression of two genes coding for subunits of RNA polymerases (*rpoC*^*RC1_0702*^, *rpoA*^*RC1_0735*^) (S21A Fig). Increased RNA polymerase synthesis is not unexpected, given that there are numerous increases in gene expression early in germination. There are also nine sigma factors that exhibit changes in expression at various times during germination (S21B Fig). Intriguingly, RC1_1842, RC1_2001, RC1_2169, and RC1_3812 exhibit reduced expression during germination while during cyst formation, these same sigma factors undergo increased expression [21]. This bifurcation in expression indicates that they may have a role in cyst development or perhaps cyst maintenance. There is also, a gene coding for sigma factor regulatory protein (*fecR*^*RC1_3844*^) involved in ferric iron transport that is upregulated early and throughout the germination process (S21B Fig).

Looking at annotated transcription factors, there are 39 that show significant downregulation and 16 that undergo increased expression throughout the germination time spectrum (S22A, S22B and S22C Fig). Notable on the list are the transcription factors RC1_1377 and RC1_3187 that show very early increases in expression at 0.5 hrs and 1.5 hrs after induction of germination. Presumably, these would be good candidates as regulators involved in early germination events. It is of note that some of these transcription factors show an opposite direction of regulation during germination versus cyst development. For example, RC1_1377 shows early ramped-up expression during germination but also a significantly ramped-down expression during cyst development (S22C Fig). This is an expression pattern expected from a master controller of either the germination or cyst development process. Later, at 4–5 hrs into germination, there is increased expression of a MucR type regulator (*RC1_2608*) and an ExpG regulator that in other species are known to regulate expression of genes involved in exopolysaccharide synthesis. These regulators are likely involved in controlling genes involved in late cell wall remodeling before emergence from the exine husk.

Regarding signal transduction, there are 12 histidine kinases (HKs) and 14 response regulators that are differentially expressed during germination (S23 Fig). The first histidine kinase to show upregulation is *RC1_0476* at 2.5 hours post induction of germination followed by HK RegB^RC1_2541^ that increases expression ~3 hrs post induction of germination (S23A Fig). RegB^RC1_2541^ is a redox monitoring HK that is known to coordinate synthesis of the energy generating pigment and protein components of the photosystem as well as respiratory electron transfer components (cytochrome oxidase, ubiquinones etc.) [56]. These energy generating processes are also synthesized at ~ 3hrs post induction of germination (Fig 5). *RC1_0688* and *RC1_3999* also show opposing regulation during cyst formation and cyst germination (S23A Fig) suggesting that they may be directly involved in regulating one or more steps of these developmental processes.

Among the seven response regulators that show upregulation, the earliest is *narL*^*RC1_1407*^, involved in nitrogen metabolism, showing significant expression at 3 hours post induction. *ctrA*^*RC1_1752*^ and *divK*^*RC1_1533*^, are also two cell cycle response regulators that also show upregulation late in germination at ~6 hours post induction. *RC1_1592*, *RC1_1779*, *RC1_2146*, and *RC1_2399* also show opposing regulations during germination and encystation and are thus good candidates for regulatory genes involved in germination (S23B Fig).

Finally, expression of the *che3* gene cluster is also ramped down as cells undergo germination (S23C Fig). The che3 signal transduction cascade is known to be involved in the formation of cyst formation, so it is not surprising that its expression is ramped down during germination [8, 10, 50].

### Conclusions

**Fig 7:**
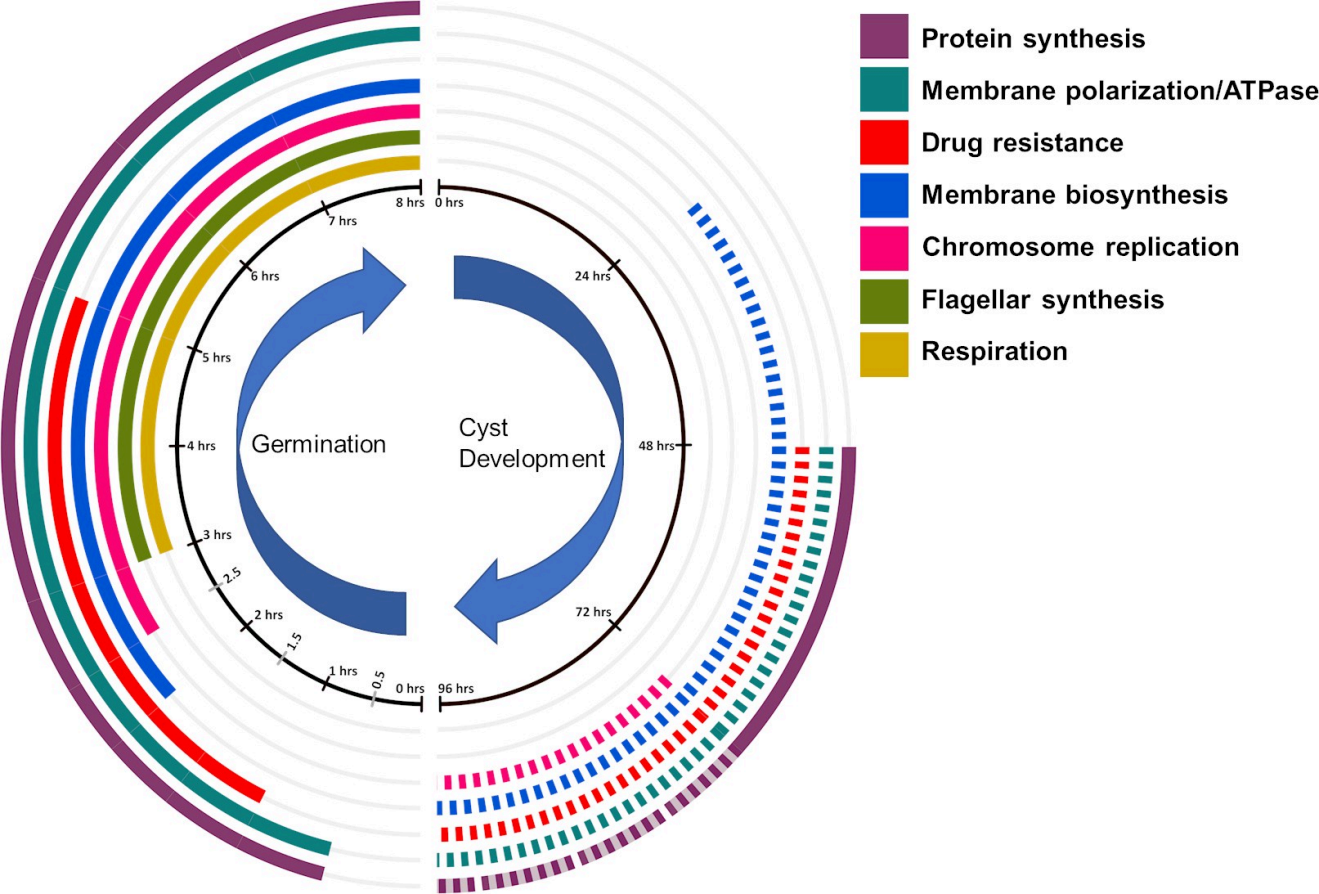
Lifecycle clock summarizing pathways and processes that are differentially regulated during the development of cysts versus cyst germination. Solid lines indicate up-regulation in expression while dotted lines indicate down-regulation. Colored transparent lines indicate periods of no changes in expression.

Microscopic morphological analysis, coupled with transcriptomics during *R*. *centenum* cyst germination, has allowed us to obtain chronological parsing of transcription events that occur during germination. Pathways and processes can be readily binned into ‘early’ (within 1.5 hours), ‘early-mid’ (1–3 hours), ‘mid-late’ (3–6 hours) and ‘late’ (6–8 hours) time frames which show that there is a distinct temporal progression of gene expression during germination (Fig 7). Distinct temporal expression changes sequentially promote the following cellular processes; (i) a significant ramp up in protein synthesis capability, (ii) concurrent early synthesis of NADH dehydrogenase that promotes production of a proton gradient, concurrent with the synthesis of ATPase that utilizes a proton gradient to generate ATP, (iii) ramped up synthesis of multi-drug exporters providing resistance to microbial toxins (antibiotics). These early events are followed by early/mid germination expression changes that promote (IV) lipid and LPS remodeling and (iv) DNA replication. Later mid/late germination events then promote (v) changes in central metabolism and the synthesis of electron transfer components that favor energy production via oxidative phosphorylation, (vi) synthesis of a photosystem followed by, (vii) synthesis of flagellar and chemotaxis components promoting motility. Throughout the germination process, there are numerous expression changes in transcription and signal transduction factors that presumably function as conductors that orchestrate observed temporal changes in gene expression.

Finally, comparative analysis of germination gene expression changes in this study with prior analysis of expression changes during cyst development reveals that there are numerous examples of refractile changes in gene expression between the germination and development of cysts. As shown in Fig 7, there is ramped-down in expression of protein synthesis machinery, chromosome replication, and lipid biosynthesis late in cyst development (Fig 7, dotted lines). In contrast, there is ramped-up expression of these same processes during cyst germination (Fig 7, solid lines). There are also numerous regulatory proteins (transcription factors, sigma subunits, signal transduction components, etc) that undergo an inverse expression pattern between cyst cells that are undergoing germination versus vegetative cells that are undergoing the development of cysts. These are potential candidate genes that may be directly involved in regulating either germination or cyst development. Future analysis of these regulatory genes should provide valuable molecular insight on how *R*. *centenum* and other Gram-negative species develop dormant cyst and how cysts undertake the process of germination.

Finally, beyond our study on the germination of a Gram-negative cyst, and studies of Gram-positive spore germination [12–14], there are very few reports on what occurs as cells emerge from dormancy. Like *R*. *centenum* cyst germination, Gram-positive spore germination also appears to involve programmed expression of different cellular processes such as cell wall remodeling, alteration of lipid composition, followed by increased gene expression involving transport, protein synthesis, and DNA replication [12–14]. The order by which individual processes undergo altered expression is slightly different between cysts and spores but it’s clear that emergence from dormancy in both of these cell types involved ordered programed events. Beyond spores and cysts, it is well known that cells in biofilms and late stationary cultures form non-replicating persister cells that exhibit significantly reduced metabolism [57–59]. Due to their low metabolism, they are often resistant to antibiotics that are designed to target cells with more active metabolism and replication [60]. Like classic dormant cysts and spores, the addition of fresh nutrients has been observed to immediately resuscitate persister cells [58]. Not unlike *R*. *centenum* cysts that prioritize ribosome synthesis early in germination, the ribosome content of persister cells are known to play a role in persister cell awakening with cells that have higher levels of ribosomes waking up first [61, 62]. This phenomenon has been attributed to the mandatory need for protein production for successful persister cell awakening [61, 62]. One long-term goal of this study, and others, is to determine if there are commonalities in processes by which different species both become dormant and also awaken from dormancy. If commonalities exist, then it may allow the development of tools to control these cellular events.

## Methods

### Bacterial strain, media, and cyst isolation

Wild type (WT) *Rhodospirillum centenum* (ATCC51521) was used throughout this study [47]. CENS medium [43] was used for routine growth, while CENS containing 8 times the nitrogen content (CENS-8XN) was used for inducing cyst formation [5].

For making cysts, a single colony of *R*. *centenum* was transferred from agar solidified CENS medium into 5mls of CENS liquid medium and then aerobically grown with shaking at 37°C. 50μL of this overnight culture was then transferred to 25mls of CENS liquid medium and grown aerobically at 37°C for 15–16 hours. OD_660_ of this culture was adjusted to 2.0 with 1ml of the OD_660_ adjusted culture spun down at 8,000 × *g* for 2 min, washed thrice with 40mM pH 7.5 KHPO_4_ buffer and resuspended in 50μL of 40mM pH 7.5 KHPO_4_ buffer. This resuspension was used to make four 5μL spots on dry CENS-8XN plates that were incubated at 37°C for 60±5 hours. Presence of a large fraction of cysts was subsequently confirmed using 100x oil immersion microscopy. For cyst isolation, residual vegetative cells were removed from cyst containing wrinkled colonies by addition of lysozyme, which selectively lyses replicative vegetative cells but not dormant cysts. For lysozyme treatment, wrinkled colonies from CENS-8XN plates were harvested after 60±5 hours of growth by resuspending approximately 3.53 × 10^6^ cells/ml in 40mM pH-7.0 KHPO_4_ buffer and subsequently pelleting cells by centrifugation at 8,000 × *g* for 2 min. The pellet was resuspended in TE buffer containing 200μg/ml of lysozyme, incubated at 37°C for 30 minutes and washed multiple times with 40mM, pH 7.0 KHPO_4_ buffer. Cysts present in the resulting pellet, were >90% enriched over vegetative cells as based on flow cytometric analyses (S1 Fig). Enriched cysts were prepared fresh before each germination assay/study.

### Microscopy

Cysts harvested from 60±5 hour colony grown on CENS-8XN plates, were inoculated on top of a 1% CENS agarose pad on a concavity slide to which a coverslip was added and sealed using VALAP [63]. This slide was incubated on a 60X oil immersion objective of a Nikon TiE inverted microscope with an objective warmer set at 37°C. The cysts on the slide were recorded based on their X, Y coordinates using NIE elements viewer software© and imaged every 5 minutes (phase contrast) for the next 15 hours. ImageJ (FIJI) was used for adding the time stamp and for stitching the images into a movie. Negative staining electron microscopy and scanning electron microscopy was undertaken as described in ref [1].

### RNA-Seq transcriptomic analysis of germination

Approximately 1.13 x 10^5^ isolated cysts/ml were inoculated into nutrient-rich CENS medium in 2L Erlenmeyer flasks and incubated at 37°C with continuous stirring on a magnetic plate. At 0 hr, a 10ml control sample was taken immediately after addition of CENS medium to cysts. 10ml samples were then taken every half an hour for the first 3rd hours and every 1 hour after that until a final sample at the 8th hour. Upon collection, samples were immediately pelleted in a cold centrifuge and resuspended in 1ml of Trizol and transferred to a lysing matrix B tube® (MP Biomedicals), lysed in a Fastprep® instrument (MP Biomedicals), and spun down at 8,000 × g. The resulting supernatant was stored at -80°C until RNA extraction.

RNA was extracted using the DirectZol^™^ RNA miniprep plus (Zymo Research) using manufacturer's instructions. Briefly, after adding an equal volume of ethanol to the Trizol^™^ lysed sample, the mix was loaded on to Zymo-Spin^™^ IIICG column, centrifuged, flow through discarded and the in-column DNase I treatment was done. After washes, total RNA eluted in nuclease-free water.

### Quality control, library preparation, and RNA sequencing

Analysis of RNA quality, cDNA library preparation, and deep sequence analysis was undertaken by the Gene Expression Center and DNA Sequencing Facility at the University of Wisconsin-Madison Biotechnology Center. Briefly, RNA concentration and quality were determined using nanodrop and Agilent 2100 bioanalyzers. Samples were checked for sharp 16S and 23S rRNA peaks before downstream processing. Ribosomal RNA from the total RNA was removed using EpiCentre® Ribo-Zero^™^ Magnetic (Bacteria) kit with an input quantity of 1.5μg total RNA. TruSeq^™^ RNA Sample Prep kit (Illumina, San Diego, CA) was used to prepare cDNA mRNA-seq libraries. Standard Illumina protocol was then used to perform single end sequencing (1X100bp) on Illumina HiSeq 2000 to a level that provided over 200-fold coverage per nucleotide of the genome.

### Raw data processing and data analysis

In-house data analysis pipeline was used for raw data processing. Briefly, Trimmomatic [64] was used to trim the reads off adaptors with low-quality reads from the ends (sliding window– 5:25; minimum read length– 40bp). Bowtie2 [65] then used to align trimmed reads to *R*. *centenum* reference genome followed by HT-seq-count [66] to detect the number of reads per gene. Overall there were over 130 million (M) (strand specific) RNA-Seq reads with >99% of these reads mapped to the *R*. *centenum* genome yielding over 200X coverage per nucleotide. This sequence data has been deposited in NCBI’s Gene Expression Omnibus [67] and are accessible through GEO Series accession number GSE132286 (https://www.ncbi.nlm.nih.gov/geo/query/acc.cgi?acc=GSE132286).

Differential expression between the 0th-hour sample, and every other time point, was then calculated using the DE-Seq2 package in R [68]. A gene was considered as a Differentially Expressed Gene (DEG) when the log_2_ fold change was ≥ ± 1.5 with a false discovery rate adjusted p-value of <0.05. The differentially expressed genes were sorted into COGs (Cluster of Orthologous Groups) using Eggnog 4.5.1 [69] and Kegg [45, 70–72] with COG X assigned to photosynthesis genes [73]. Final manipulation with the output file (Tab-delimited text file) was done using Microsoft Excel^™^.

### qRT-PCR validation of RNA-seq results

Five genes that were differentially expressed in the RNA-seq data sets were chosen (RC1_3511, RC1_1228, RC1_0470, RC1_0099, RC1_0097) and independently analyzed for expression using qRT-PCR. Briefly, RNA extracted from samples collected during specific timepoints of germination (0hr, 0.5hr, 2.5hr and 6 hr.) for 3 biological replicates were reverse transcribed using Takara PrimeScript^TM^ RT Master Mix. TB Green Premix Ex Taq^TM^ II with ROX was used to perform qRT-PCR in an Applied Biosystems StepOne Plus instrument. The thermocycling conditions used were as follows: 95°C for 30 seconds (1 cycle), 95°C for 5 seconds and 60°C for 30 seconds (40 cycles) followed by a melt curve stage. Bestkeeper analysis and previous qPCR validations have identified RC1_1500 to be an ideal housekeeping gene in *R*. *centenum* [74, 75] and the same has been used here for normalization. Log_2_fold change with respect to time 0 for each gene and timepoint were calculated and plotted against the log^2^ fold change of the RNA-seq data (S2 Fig).

## Supporting information

S1 Movie A video showing cyst germination on a 1% CENS agarose pad incubated at 37°C over an 8 hour time period. Cyst germination was imaged every 5 minutes the with recorded images stitched into a movie.
(AVI)

S1 Table A spread sheet listing 1667 genes that undergo differential expression between the 0th-hour sample, and every other time point, was then calculated using the DE-Seq2 package in R [62]. A gene was considered as a Differentially Expressed Gene (DEG) when the log_2_ fold change was ≥ ± 1.5 with a false discovery rate adjusted p-value of <0.05.
(XLSX)

S1 Fig Representative flow cytometry data showing the enrichment of cysts for germination studies. A) Tight flow cytometry clustering of a vegetative cell culture. Due to their uniform size, shape and complexity vegetative cells have small forward and side scatters. B) Cells from a wrinkled colony grown on a CENS 8XN plate contain a mixture of vegetative (37.9%) and cyst (61.7%) cells. Cysts form a comet tail on flow cytometric data due to their variations in size, shape and internal complexity. C) An enrichment of cysts (97.8% enriched) obtained after treatment of cells in B with lysozyme.
(TIFF)

S2 Fig Correlation validation plot between RNA-seq data and qRT-PCR data sets. Relative quantification for 5 genes that were DEG in the RNA-seq data set were validated by qRT-PCR amplification at 4 time points (0hr, 0.5hr, 2.5hr and 6hr) with normalization using the housekeeping gene RC1_1500. For each gene and time point, log_2_ fold change with respect to the 0^th^ hour was calculated. The qRT-PCR data sets were plotted against expression changes obtained from the same genes at the same time from RNA-seq data sets.
(TIFF)

S3 Fig Differentially expressed genes that are binned into COGS. Figure shows the number of upregulated (green) and down regulated (red) genes in each COG. Highest number of genes were found in the bins for ‘Function unknown’ and ‘No orthologs found’ which are presented as a separate inset to help with data visualization.
(TIFF)

S4 Fig Comparison of transcriptomics during encystation (cyst formation) and cyst germination. A) Venn diagram showing overlap between genes showing expression changes during cyst germination and cyst formation as measured using RNA-seq. B) Common genes of both developmental processes sorted into similarly (orange) and opposingly (pink) regulated groups and then organized into COGS. C) Genes for which ‘No orthologs’ were found and genes classified as ‘Function unknown’ as classified by Eggnog.
(TIFF)

S5 Fig Expression heat maps of genes involved in protein synthesis. A) and B) show increase expression of genes for large and small ribosomal subunits early in germination, respectively. C) shows changes in expression of ribosomal initiation, elongation and release factors. D) shows expression changes of genes involved in protein folding. E) shows early expression of *ftsY*, coding for a component of the signal recognition system that targets integral membrane proteins to the membrane. Color of boxes areas noted in S3 Fig and the numbers representing a log_2_ fold change.
(TIFF)

S6 Fig Early expression of protein complexes involved in formation of a membrane potential and use of this potential for energy formation. A) early expression of pyruvate dehydrogenase that generates NADH as a product. B) early expression of NADH dehydrogenase (complex I) that utilizes NADH to form a membrane potential and (C) early expression of ATP synthase (complex V) that utilizes a membrane potential for ATP production. Color of boxes are as noted in S3 Fig and the numbers represent the log_2_ fold change.
(TIFF)

S7 Fig Heat maps of multidrug transporters show early expression during cyst germination. There is also opposing regulation during cyst germination (left heat map) versus cyst formation (right heat map) for several drug export genes. Color of boxes are as noted in S3 Fig and the numbers represent the log_2_ fold change.
(TIFF)

S8 Fig Heat map of gene expression changes that affect fatty acid (A) and peptidoglycan (B) biosynthesis. Color of boxes are as noted in S3 Fig and the numbers represent the log_2_ fold change.
(TIFF)

S9 Fig Numerous genes involved in biosynthesis of the lipopolysaccharide lipid A core (A) and the O-antigen (B) that undergo a ramp up in expression. This is contrasted by decreased expression of genes involved in cell wall recycling (C) and genes involved in synthesis of exopolysaccharide precursors involved in cyst cell wall exine synthesis (D). Color of boxes are as noted in S3 Fig and the numbers represent the log_2_fold change.
(TIFF)

S10 Fig Heat map of genes involved in chromosome replication and partitioning (A) and coding for enzymes involved in nucleotide metabolism (B & C) and pyrophosphatase (D). Color of boxes are as noted in S3 Fig and the numbers represent the log_2_ fold change.
(TIFF)

S11 Fig Heat map of genes coding for electron transfer components involved in respiratory oxidative phosphorylation. Many genes coding for enzymes in the TCA cycle (A and B) exhibit increased expression midway during germination. Components of the respiratory electron transport chain (C, D and E) also undergo increased expression during a similar period of germination. Color of boxes are as noted in S3 Fig and the numbers represent the log_2_ fold change.
(TIFF)

S12 Fig Reduction of anaerobic fermentation processes midway/late in germination. (A & B), reduction in expression of lactate dehydrogenase and acetyl-CoA synthetase that are involved in production of lactate and acetate, respectively. (C) reduced expression of numerous enzymes involved in fatty acid β-oxidation. (D) Reduced expression of enzymes involved in polyhydroxybutyrate (PHB) synthesis during cyst germination. The left heat maps are from RNA-seq data obtained during cyst germination while the right heat maps are derived from independent RNA-seq data during cyst formation. Each of these processes are undergo inverse expression patterns during these different stages of cyst development. Color of boxes are as noted in S3 Fig and the numbers represent the log_2_ fold change.
(TIFF)

S13 Fig Heat map of gene expression changes for genes involved in FeS and amino acid biosynthesis. In some cases, there are also expression changes observed during cyst development is shown on the right most heat maps. Color of boxes are as noted in S3 Fig and the numbers represent the log_2_ fold change.
(TIFF)

S14 Fig Heat map of genes involved in synthesis of the bacteriochlorophyll, light harvesting and reaction center components of the photosystem (A) as well as several genes involved in the heme and bacteriochlorophyll common trunk of the tetrapyrrole pathway (B). Color of boxes are as noted in S3 Fig and the numbers represent the log_2_ fold change.
(TIFF)

S15 Fig Heat map showing expression profiles of polar (A) and lateral (B) flagellar genes. Operons coding for flagellar gene clusters (FGC) are indicated to the left of the heat maps. (C) is a heat map of the Che2 gene cluster that is involved in the synthesis of lateral flagella and (D) is a heat map of the Che1 gene cluster that is involved in chemotaxis and phototaxis. Color of boxes are as noted in S3 Fig and the numbers represent the log_2_ fold change.
(TIFF)

S16 Fig Heat map showing expression profiles of R body genes that goes down during germination (left) and up during cyst formation (right). Color of boxes are as noted in S3 Fig and the numbers represent the log_2_ fold change.
(TIFF)

S17 Fig Heatmaps show the expression profiles of various transport systems expressed at the early time point of germination. (A) Zinc/manganese/iron transport systems. (B) ABC transporters of phosphate. (C) Efflux transporters for monovalent cations. (D) K^+^ stimulated pyrophosphate energized sodium pump. Color of boxes are as noted in S3 Fig and the numbers represent the log_2_ fold change.
(TIFF)

S18 Fig Heatmaps show the expression profiles of the lipopolysaccharide (A) and molybdenum (B) transport systems that are expressed at the early to middle time points of germination. Lipopolysaccharide expression is induced during cyst germination (left heat maps) and reduced during cyst formation (right heat maps). Color of boxes are as noted in S3 Fig and the numbers represent the log_2_ fold change.
(TIFF)

S19 Fig Heatmaps show the expression profiles of transport systems expressed at the middle to late time points of germination. (A) ABC transporters specific for sugar transport. (B) Branched-chain amino acid transport. (C) Putrescine transport. (D) Oligopeptide transport. (E) Potassium transport. (F) Active transport of potassium. (G) Copper transport. Color of boxes are as noted in S3 Fig and the numbers represent the log_2_ fold change.
(TIFF)

S20 Fig Heatmaps show the middle to late expression profiles of *exbB* and *exbD* that code for TonB receptor components involved in transport across the inner membrane (A) and various TonB dependent outer membrane receptors (B). Color of boxes are as noted in S3 Fig and the numbers represent the log_2_ fold change.
(TIFF)

S21 Fig Heatmaps show the expression profiles of holo-enzyme RNA polymerase subunits (A) and sigma factors (B). Color of boxes are as noted in S3 Fig and the numbers represent the log_2_ fold change.
(TIFF)

S22 Fig Time line showing the point of significant expression changes of every differentially regulated transcription factor in this dataset (A). Heatmaps showing the expression profiles of down regulated (B) and upregulated (C) transcription factors. Heat maps on the left side of B and D are derived from RNA-seq data sets during cyst germination while the heat maps on the right side are from RNA-seq data sets during cyst formation. Color of boxes are as noted in S3 Fig and the numbers represent the log_2_ fold change.
(TIFF)

S23 Fig Heatmaps show the expression profiles of histidine kinases (A), response regulators (B) and the che3 gene cluster involved in cyst development (C). Heat maps on the left side are derived from RNA-seq data sets during cyst germination while the heat maps on the right side are from RNA-seq data sets during cyst formation. Color of boxes are as noted in S3 Fig and the numbers represent the log_2_ fold change.
(TIFF)

## References

[1] Berleman JE, Bauer CE. Characterization of cyst cell formation in the purple photosynthetic bacterium Rhodospirillum centenum. Microbiology, 2004; 150: 383–390. 10.1099/mic.0.26846-0 14766916

[2] Socolofsky MD, Wyss O. Resistance of the Azotobacter cyst. J Bacteriol, 1962; 84: 119–124. 13914732 10.1128/jb.84.1.119-124.1962 PMC277776

[3] Higgins D, Dworkin J. Recent progress in Bacillus subtilis sporulation. FEMS Microbiol Rev, 2012; 36: 131–148. 10.1111/j.1574-6976.2011.00310.x 22091839 PMC3237856

[4] Narula J, Fujita M, Igoshin OA. Functional requirements of cellular differentiation: lessons from Bacillus subtilis. Curr Opin Microbiol, 2016 34: 38–46. 10.1016/j.mib.2016.07.011 27501460

[5] Marden JN, et al, Cyclic GMP controls Rhodospirillum centenum cyst development. Mol Microbiol, 2011; 79: 600–615. 10.1111/j.1365-2958.2010.07513.x 21214648 PMC4273943

[6] Roychowdhury S, Dong Q, Bauer CE. DNA binding properties of a cGMP binding CRP homolog that controls development of metabolically dormant cysts of Rhodospirillum centenum. Microbiology, 2015; 161: 2256–2264. 10.1099/mic.0.000172 26362215 PMC4806592

[7] Berleman JE, Hasselbring BM, Bauer CE. Hypercyst mutants in Rhodospirillum centenum identify regulatory loci involved in cyst cell differentiation. J Bacteriol, 2004; 186: 5834–5841. 10.1128/JB.186.17.5834-5841.2004 15317789 PMC516826

[8] Berleman JE, Bauer CE. Involvement of a Che-like signal transduction cascade in regulating cyst cell development in Rhodospirillum centenum. Mol Microbiol, 2005; 56:1457–1466. 10.1111/j.1365-2958.2005.04646.x 15916598

[9] Dong Q, Fang M, Roychowdhury S, Bauer CE. Mapping the CgrA regulon of Rhodospirillum centenum reveals a hierarchal network controlling Gram-negative cyst development. BMC Genomics, 2015; 16:1066 10.1186/s12864-015-2248-z 26673205 PMC4681086

[10] He K, Marden JN, Quardokus EM, Bauer CE. Phosphate flow between hybrid histidine kinases CheA3 and CheS3 controls Rhodospirillum centenum cyst formation. PLOS Genetics, 2013; 9: e1004002 10.1371/journal.pgen.1004002 24367276 PMC3868531

[11] Din N, Shoemaker CJ, Akin KL, Frederick C, Bird TH. Two putative histidine kinases are required for cyst formation in Rhodospirillum centenum. Arch Microbiol, 2011; 193: 209–222. 10.1007/s00203-010-0664-7 21184217

[12] Setlow P. Germination of Spores of Bacillus Species: What We Know and Do Not Know. J Bacteriol, 2014; 196: 1297–1305. 10.1128/JB.01455-13 24488313 PMC3993344

[13] Setlow P, Wang S, Li YQ. Germination of Spores of the Orders Bacillales and Clostridiales. Annu Rev Microbiol, 2017; 71: 459–477. 10.1146/annurev-micro-090816-093558 28697670

[14] Driks A, Eichenberger P.,*The Bacterial Spore*: *From Molecules to Systems*. 2016; Washington, DC: ASM Press.

[15] Bhattacharjee D, McAllister KN, Sorg JA. Germinants and their receptors in Clostridia. J Bacteriol, 2016; 198: 2767–2775. 10.1128/JB.00405-16 27432831 PMC5038010

[16] Lin LP, Pankratz S, Sadoff HL. Ultrastructural and physiological changes occurring upon germination and outgrowth of Azotobacter vinelandii cysts. J bacteriol 1978; 135: 641–646. 681284 10.1128/jb.135.2.641-646.1978 PMC222425

[17] Wyss O, Neumnn MG, Socolofsky MD. Development and germination of the Azotobacter cyst. J biophys biochem cytology, 1961; 10: 555–565. 10.1083/jcb.10.4.555 PMC2225101 13787014

[18] Loperfido B, Sadoff HL. Germination of Azotobacter vinelandii Cysts: Sequence of Macromolecular Synthesis and Nitrogen Fixation. J Bacteriol, 1973; 113: 841–846. 4690966 10.1128/jb.113.2.841-846.1973 PMC285299

[19] Stevenson LH, Socolofsky MD. 1966. Cyst formation and poly-b-hydroxybutyric acid accumulation in Azotobacter. J Bact., 1966; 91: 304–310. 5903098 10.1128/jb.91.1.304-310.1966 PMC315949

[20] Hitchins AD, Gould GE, Hurst A. The swelling of bacterial spores during germination and outgrowth. J Gen Microbiol, 1963; 30: 445–453. 10.1099/00221287-30-3-445 13954805

[21] Dong Q, Bauer CE. Transcriptome analysis of cyst formation in Rhodospirillum centenum reveals large global changes in expression during cyst development. BMC Genomics, 2015 16: 68 10.1186/s12864-015-1250-9 25758168 PMC4340629

[22] Sinai L, Rosenberg A, Smith Y, Segev E, Ben-Yehuda. The molecular timeline of a reviving bacterial spore. Molecular Cell, 2015; 57: 695–707. 10.1016/j.molcel.2014.12.019 25661487 PMC4339302

[23] Koch HG, Moser M, Muller M. Signal recognition particle-dependent protein targeting, universal to all kingdoms of life. Rev Physiol Biochem Pharmacol, 2003; 146: 55–94. 10.1007/s10254-002-0002-9 12605305

[24] Wild K, Rosendal KR, Sinning I. A structural step into the SRP cycle. Mol Microbiol, 2004 53: 357–363. 10.1111/j.1365-2958.2004.04139.x 15228518

[25] Keijser BJ, et al, Analysis of temporal gene expression during Bacillus subtilis spore germination and outgrowth. J Bacteriol, 2007; 189: 3624–3634. 10.1128/JB.01736-06 17322312 PMC1855883

[26] Reusch RN, Sadoff HL. Lipid metabolism during encystment of Azotobacter vinelandii. J Bacteriol, 1981; 145: 889–895. 7462162 10.1128/jb.145.2.889-895.1981 PMC217195

[27] Reusch RN, Sadoff HL. Novel lipid components of the Azotobacter vinelandii cyst membrane. Nature, 1983; 302: 268–270. 10.1038/302268a0 6835364

[28] O'Leary WM. The fatty acids of bacteria. Bacteriological Reviews, 1962; 26: 421–447. 16350179 10.1128/br.26.4.421-447.1962 PMC441163

[29] Touz ET, Mengin-Lecreulx D. Undecaprenyl phosphate synthesis. EcoSal Plus, 2008; 3. 10.1128/ecosalplus.4.7.1.7 26443724

[30] Park JT, Uehara T. How bacteria consume their own exoskeletons (turnover and recycling of cell wall peptidoglycan). Microbiol Mol Biol Rev, 2008; 72: 211–227. 10.1128/MMBR.00027-07 18535144 PMC2415748

[31] Bignell C, Thomas CM. The bacterial ParA-ParB partitioning proteins.J Biotechnol, 2001; 91: 1–34. 10.1016/s0168-1656(01)00293-0 11522360

[32] Cooper S, Helmstetter CE. Chromosome replication and the division cycle of Escherichia coli B/r. J Mol Biol, 1968; 31: 519–540. 10.1016/0022-2836(68)90425-7 4866337

[33] Fossum S, Crooke E, Skarstad K. Organization of sister origins and replisomes during multifork DNA replication in Escherichia coli. EMBO J, 2007; 26: 4514–4522. 10.1038/sj.emboj.7601871 17914458 PMC2063475

[34] Setlow P. Deoxyribonucleic acid synthesis and deoxynucleotide metabolism during bacterial spore germination. J Bacteriol, 1973; 114: 1099–1107. 4197265 10.1128/jb.114.3.1099-1107.1973 PMC285370

[35] Chakrabarty AM, Nucleoside diphosphate kinase: role in bacterial growth, virulence, cell signalling and polysaccharide synthesis. Mol Microbiol, 1998; 28: 875–882. 10.1046/j.1365-2958.1998.00846.x 9663675

[36] Setlow P. Percent charging of transfer ribonucleic acid and levels of ppGpp and pppGpp in dormant and germinated spores of Bacillus megaterium. J Bacteriol, 1974; 118: 1067–1074. 4208410 10.1128/jb.118.3.1067-1074.1974 PMC246857

[37] Chen J, et al, Pyrophosphatase is essential for growth of Escherichia coli. Journal of bacteriology, 1990; 172: 5686–5689. 10.1128/jb.172.10.5686-5689.1990 2170325 PMC526883

[38] Heinonen JK. Biological role of inorganic pyrophosphate. 2001; Boston: Kluwer Academic Publishers viii, 250 p.

[39] Poon WW, et al, Identification of Escherichia coli ubiB, a gene required for the first monooxygenase step in ubiquinone biosynthesis. J Bacteriol, 2000; 182: 5139–5146. 10.1128/jb.182.18.5139-5146.2000 10960098 PMC94662

[40] Setlow P, Primus G. Protein metabolism during germination of Bacillus megaterium spores. I. Protein synthesis and amino acid metabolism. J Biol Chem, 1975; 250: 623–630. 803494

[41] Kiyasu T, et al, Contribution of cysteine desulfurase (NifS protein) to the biotin synthase reaction of Escherichia coli. J Bacteriol, 2000; 182: 2879–2885. 10.1128/jb.182.10.2879-2885.2000 10781558 PMC101998

[42] Zheng L, White RH, Cash VL, Jack RF, Dean DR. Cysteine desulfurase activity indicates a role for NIFS in metallocluster biosynthesis. Proc Natl Acad Sci, 1993; 90: 2754–2758. 10.1073/pnas.90.7.2754 8464885 PMC46174

[43] Nickens D, Fry CJ, Ragatz L, Bauer CE, Gest H. Biotype of the purple nonsulfur photosynthetic bacterium, Rhodospirillum centenum. Arch Microbiol, 1996; 165: 91–96. 10.1007/BF00262196 7710317

[44] Berleman JE, Bauer CE A che-like signal transduction cascade involved in controlling flagella biosynthesis in Rhodospirillum centenum. Mol Microbiol, 2005; 55:1457–1466. 10.1111/j.1365-2958.2005.04489.x 15720548

[45] Lu YK, et al, Metabolic flexibility revealed in the genome of the cyst-forming alpha-1 proteobacterium Rhodospirillum centenum. BMC Genomics, 2010; 11: 325 10.1186/1471-2164-11-325 20500872 PMC2890560

[46] McClain J, Rollo DR, Rushing BG, Bauer CE. Rhodospirillum centenum utilizes separate motor and switch components to control lateral and polar flagellum rotation. J Bacteriol, 2002; 184; 2429–2438. 10.1128/JB.184.9.2429-2438.2002 11948156 PMC134980

[47] Ragatz L, Jiang Z-Y, Bauer C, Gest H. Macroscopic phototactic behavior of the purple photosynthetic bacterium Rhodospirillum centenum. Arch Microbiol, 1995 163; 1–6. 10.1007/bf00262196 7710317

[48] Jiang Z-Y, Bauer CE. Analysis of a chemotaxis operon from Rhodospirillum centenum. J Bacteriol, 1997; 179: 5712–5719. 10.1128/jb.179.18.5712-5719.1997 9294426 PMC179458

[49] Jiang Z-Y, Gest H, Bauer CE. Chemosensory and photosensory perception in purple photosynthetic bacteria utilize common signal transduction components. J Bacteriol, 1997; 179: 5720–5727. 10.1128/jb.179.18.5720-5727.1997 9294427 PMC179459

[50] He K, Dragnea V, Bauer CE. Adenylate charge regulates sensor kinase CheS3 to control cyst formation in Rhodospirillum centenum. MBio, 2015; 6: e00546–15. 10.1128/mBio.00546-15 25944862 PMC4436063

[51] Pond FR., et al, R-body-producing bacteria. Microbiological Reviews, 1989; 53: 25–67. 2651865 10.1128/mr.53.1.25-67.1989 PMC372716

[52] Setlow B, Wahome PG, and Setlow P. Release of small molecules during germination of spores of Bacillus Species. Journal of bacteriology, 2008; 190: 4759–4763. 10.1128/JB.00399-08 18469112 PMC2446799

[53] Hamamoto T, Hashimoto M, Hino M, Kitada M, Seto Y, Kudo T, Horikoshi K. Characterization of a gene responsible for the Na+/H+ antiporter system of alkalophilic Bacillus species strain C-125. Molecular Microbiology, 1994; 14: 939–946. 10.1111/j.1365-2958.1994.tb01329.x 7715455

[54] Hiramatsu T, Kodama K, Kuroda T, Mizushima T, Tsuchiya T. A putative multisubunit Na+/H+ antiporter from Staphylococcus aureus. J Bacteriol, 1998; 180: 6642–6648. 9852009 10.1128/jb.180.24.6642-6648.1998 PMC107768

[55] Baykov AA, et al, Pyrophosphate-fueled Na+ and H+ transport in prokaryotes. Microbiol Mol Biol Rev, 2013; 77: 267–276. 10.1128/MMBR.00003-13 23699258 PMC3668671

[56] Wu J, Dragnea V, Bauer CE. Redox responding sensor kinases, in Two-component systems in bacteria, Gross R. and Beier D., Editors. 2012, Horizon Scientific Press p. 41–56.

[57] Hobby G.L., Meyer K., and Chaffee E. Observations on the mechanism of action of penicillin. Exp. Biol. Med. 1942; 50, 281–285.

[58] Joers Jõers A., Kaldalu N., and Tenson T. The frequency of persisters in Escherichia coli reflects the kinetics of awakening from dormancy. J. Bacteriol. 2010; 192, 3379–3384. 10.1128/JB.00056-10 20435730 PMC2897658

[59] Van den Bergh B., Fauvart M., and Michiels J. Formation, physiology, ecology, evolution and clinical importance of bacterial persisters. FEMS Microbiol. Rev. 2017; 41, 219–251. 10.1093/femsre/fux001 28333307

[60] Roberts M. E. and Stewart P. S., Modelling protection from antimicrobial agents in biofilms through the formation of persister cells. Microbiology 2005 151: p. 75–80 10.1099/mic.0.27385-0 15632427

[61] Wood T.K., Song S., and Yamasaki R., Ribosome dependence of persister cell formation and resuscitation. J Microbiol, 2019 57(3): p. 213–219. 10.1007/s12275-019-8629-2 30806978

[62] Song S. and Wood T.K., Persister cells resuscitate via ribosome modification by 23S rRNA pseudouridine synthase RluD. Environ Microbiol, 2019 10.1111/1462-2920.14828 31608580

[63] Fischer AH, et al, Mounting live cells attached to coverslips for microscopy. Cold Spring Harbor Protocols, 2008; 2008: pdb.prot4927. 10.1101/pdb.prot4927 21356764

[64] Bolger AM, Lohse M, Usadel B. Trimmomatic: a flexible trimmer for Illumina sequence data. Bioinformatics, 2014 30: 2114–2120. 10.1093/bioinformatics/btu170 24695404 PMC4103590

[65] Langmead B, et al, Ultrafast and memory-efficient alignment of short DNA sequences to the human genome. Genome Biol, 2009; 10: p. R25 10.1186/gb-2009-10-3-r25 19261174 PMC2690996

[66] Anders S, Pyl PT, Huber W. HTSeq—a Python framework to work with high-throughput sequencing data. Bioinformatics (Oxford, England), 2015; 31: 166–169. 10.1093/bioinformatics/btu638 PMC4287950 25260700

[67] Edgar R, Domrachev M, Lash AE. Gene expression omnibus: NCBI gene expression and hybridization array data repository. Nucleic Acids Res, 2002; 30: 207–210. 10.1093/nar/30.1.207 11752295 PMC99122

[68] Love MI, Huber W, Anders S. Moderated estimation of fold change and dispersion for RNA-seq data with DESeq2. Genome Biol, 2014; 15: 550 10.1186/s13059-014-0550-8 25516281 PMC4302049

[69] Huerta-Cepas J, et al, eggNOG 4.5: a hierarchical orthology framework with improved functional annotations for eukaryotic, prokaryotic and viral sequences. Nucleic Acids Research, 2015; 44: D286–D293. 10.1093/nar/gkv1248 26582926 PMC4702882

[70] Kanehisa M, Goto S. KEGG: kyoto encyclopedia of genes and genomes. Nucleic Acids Res, 2000; 28: 27–30. 10.1093/nar/28.1.27 10592173 PMC102409

[71] Kanehisa M, et al, KEGG: new perspectives on genomes, pathways, diseases and drugs. Nucleic Acids Res, 2017; 45: D353–d361. 10.1093/nar/gkw1092 27899662 PMC5210567

[72] Kanehisa M, et al, New approach for understanding genome variations in KEGG. Nucleic Acids Res, 2019; 47: D590–d595. 10.1093/nar/gky962 30321428 PMC6324070

[73] Kumka JE, Bauer CE. Analysis of the FnrL regulon in Rhodobacter capsulatus reveals limited regulon overlap with orthologues from Rhodobacter sphaeroides and Escherichia coli. BMC Genomics, 2015; 16: 895 10.1186/s12864-015-2162-4 26537891 PMC4634722

[74] Roychowdhury S., Dong Q., and Bauer C.E., DNA-binding properties of a cGMP-binding CRP homologue that controls development of metabolically dormant cysts of Rhodospirillum centenum. Microbiology, 2015 161(11): p. 2256–2264. 10.1099/mic.0.000172 26362215 PMC4806592

[75] Pfaffl M.W., et al, Determination of stable housekeeping genes, differentially regulated target genes and sample integrity: BestKeeper—Excel-based tool using pair-wise correlations. Biotechnol Lett, 2004 26(6): p. 509–15. 10.1023/b:bile.0000019559.84305.47 15127793

