# Supplementary figures and images for: Evidence of defined temporal expression patterns that lead a gram-negative cell out of dormancy
Source: PLoS Genet. 2020 Mar 23;16(3):e1008660. doi: 10.1371/journal.pgen.1008660 (PMC7117780; doi:10.1371/journal.pgen.1008660)

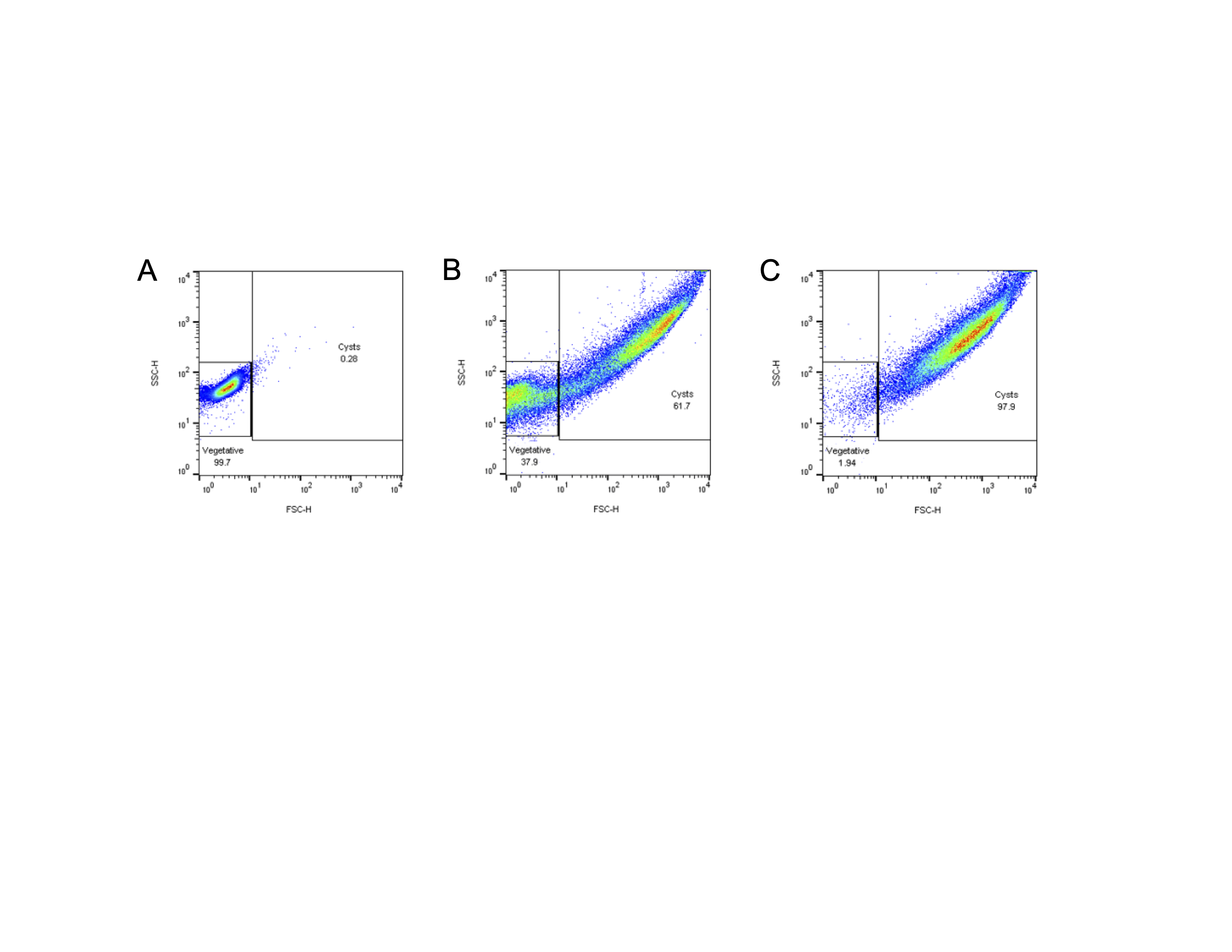

Supplement: S1 Fig — A) Tight flow cytometry clustering of a vegetative cell culture. Due to their uniform size, shape and complexity vegetative cells have small forward and side scatters. B) Cells from a wrinkled colony grown on a CENS 8XN plate contain a mixture of vegetative (37.9%) and cyst (61.7%) cells. Cysts form a comet tail on flow cytometric data due to their variations in size, shape and internal complexity. C) An enrichment of cysts (97.8% enriched) obtained after treatment of cells in B with lysozyme. (TIFF) [file pgen.1008660.s003.tiff]

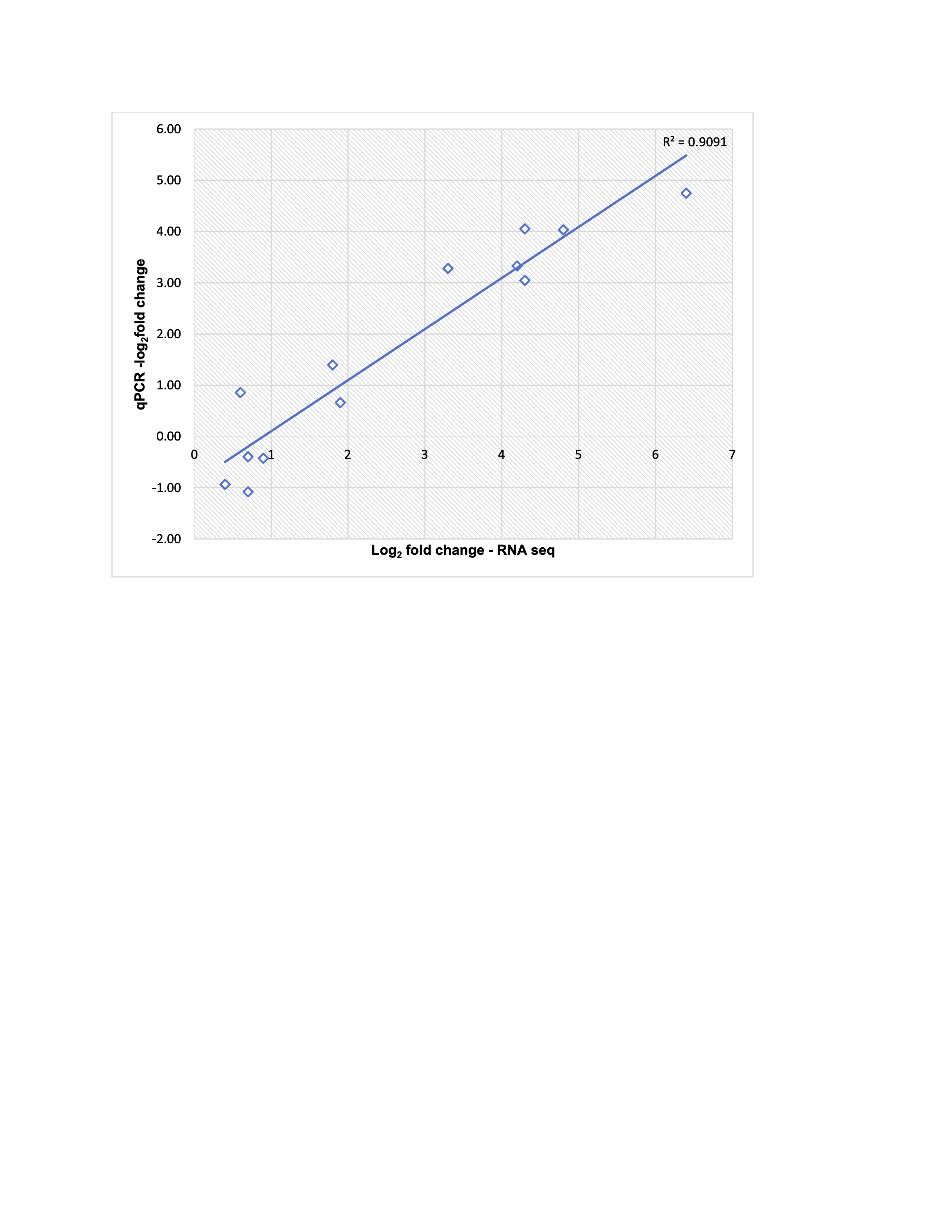

Supplement: S2 Fig — Relative quantification for 5 genes that were DEG in the RNA-seq data set were validated by qRT-PCR amplification at 4 time points (0hr, 0.5hr, 2.5hr and 6hr) with normalization using the housekeeping gene RC1_1500. For each gene and time point, log2 fold change with respect to the 0th hour was calculated. The qRT-PCR data sets were plotted against expression changes obtained from the same genes at the same time from RNA-seq data sets. (TIFF) [file pgen.1008660.s004.tiff]

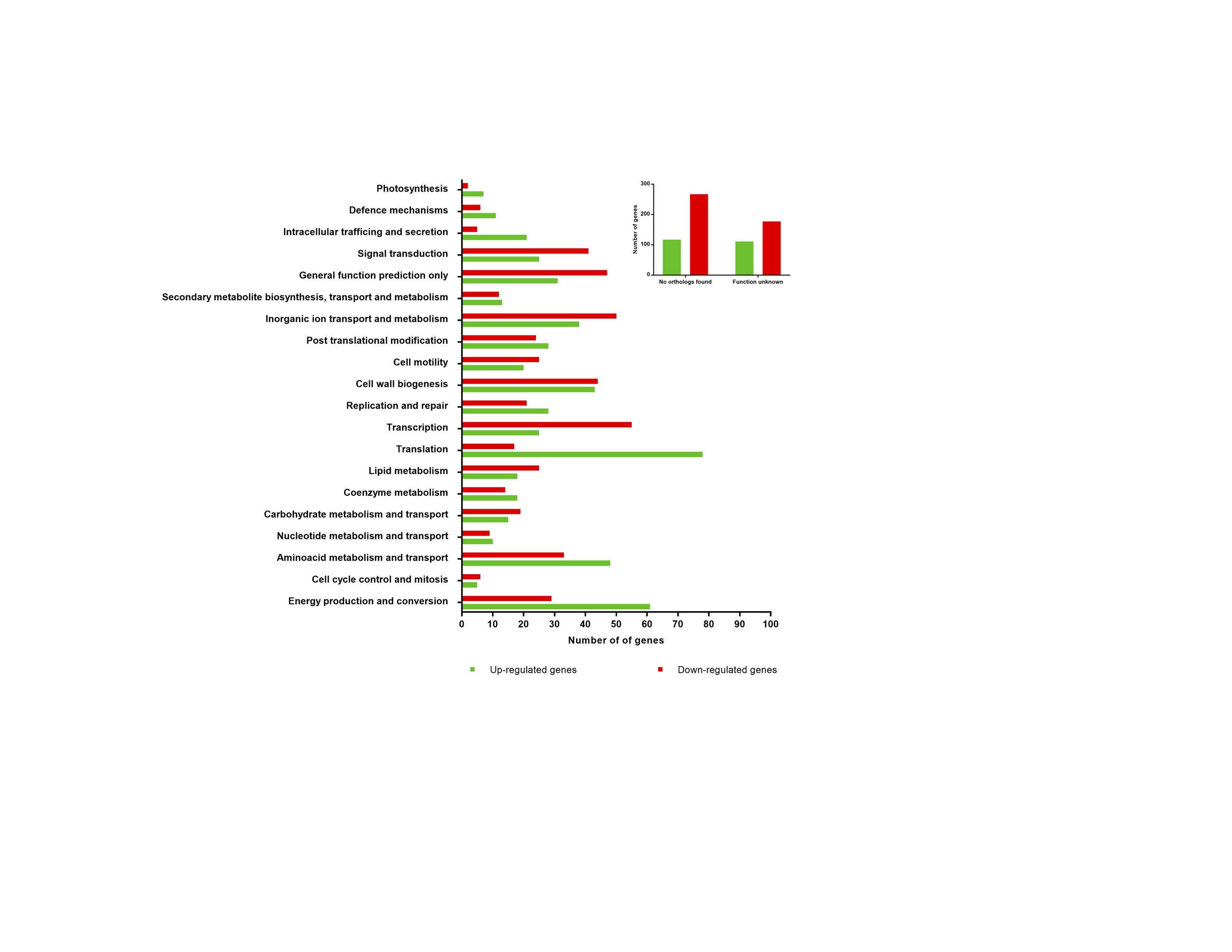

Supplement: S3 Fig — Figure shows the number of upregulated (green) and down regulated (red) genes in each COG. Highest number of genes were found in the bins for ‘Function unknown’ and ‘No orthologs found’ which are presented as a separate inset to help with data visualization. (TIFF) [file pgen.1008660.s005.tiff]

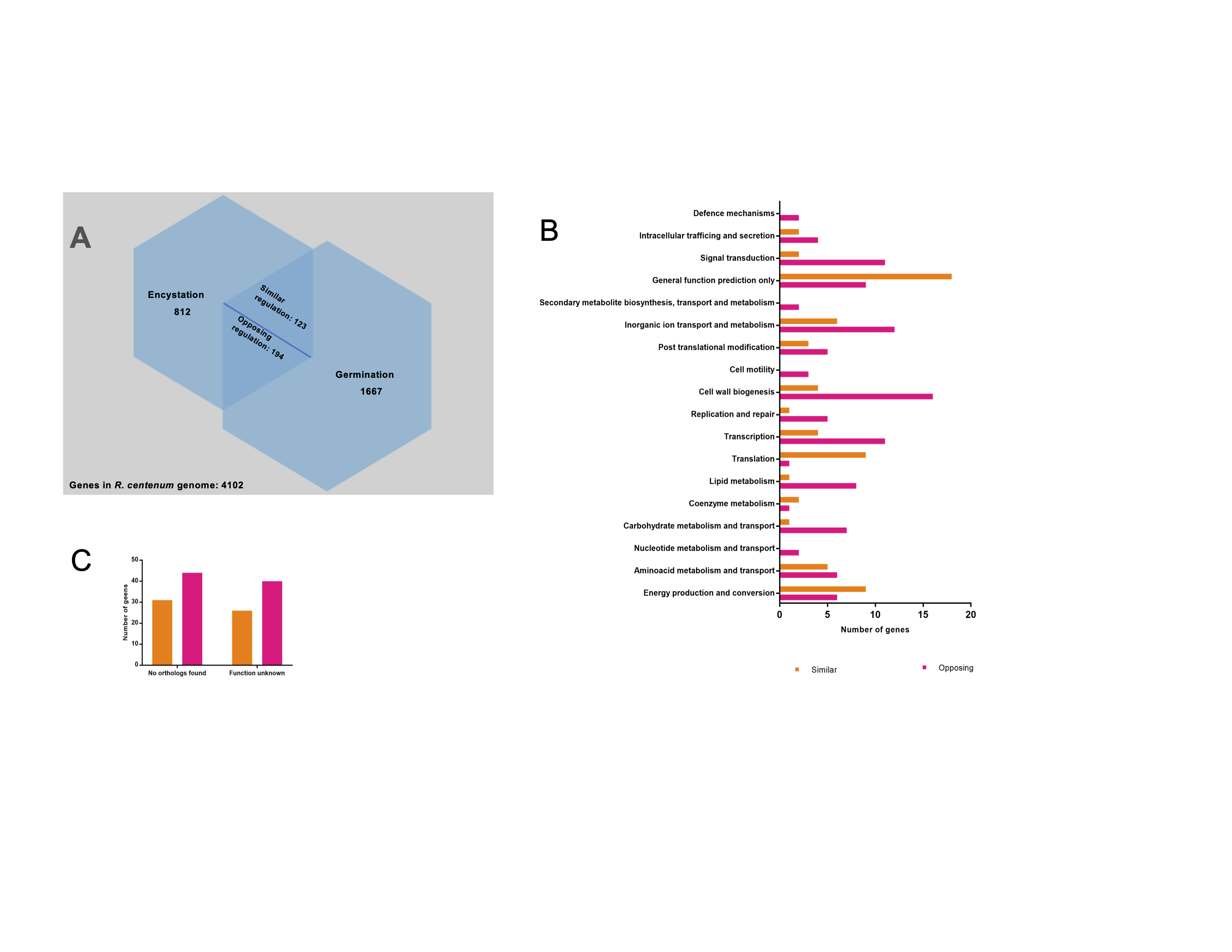

Supplement: S4 Fig — A) Venn diagram showing overlap between genes showing expression changes during cyst germination and cyst formation as measured using RNA-seq. B) Common genes of both developmental processes sorted into similarly (orange) and opposingly (pink) regulated groups and then organized into COGS. C) Genes for which ‘No orthologs’ were found and genes classified as ‘Function unknown’ as classified by Eggnog. (TIFF) [file pgen.1008660.s006.tiff]

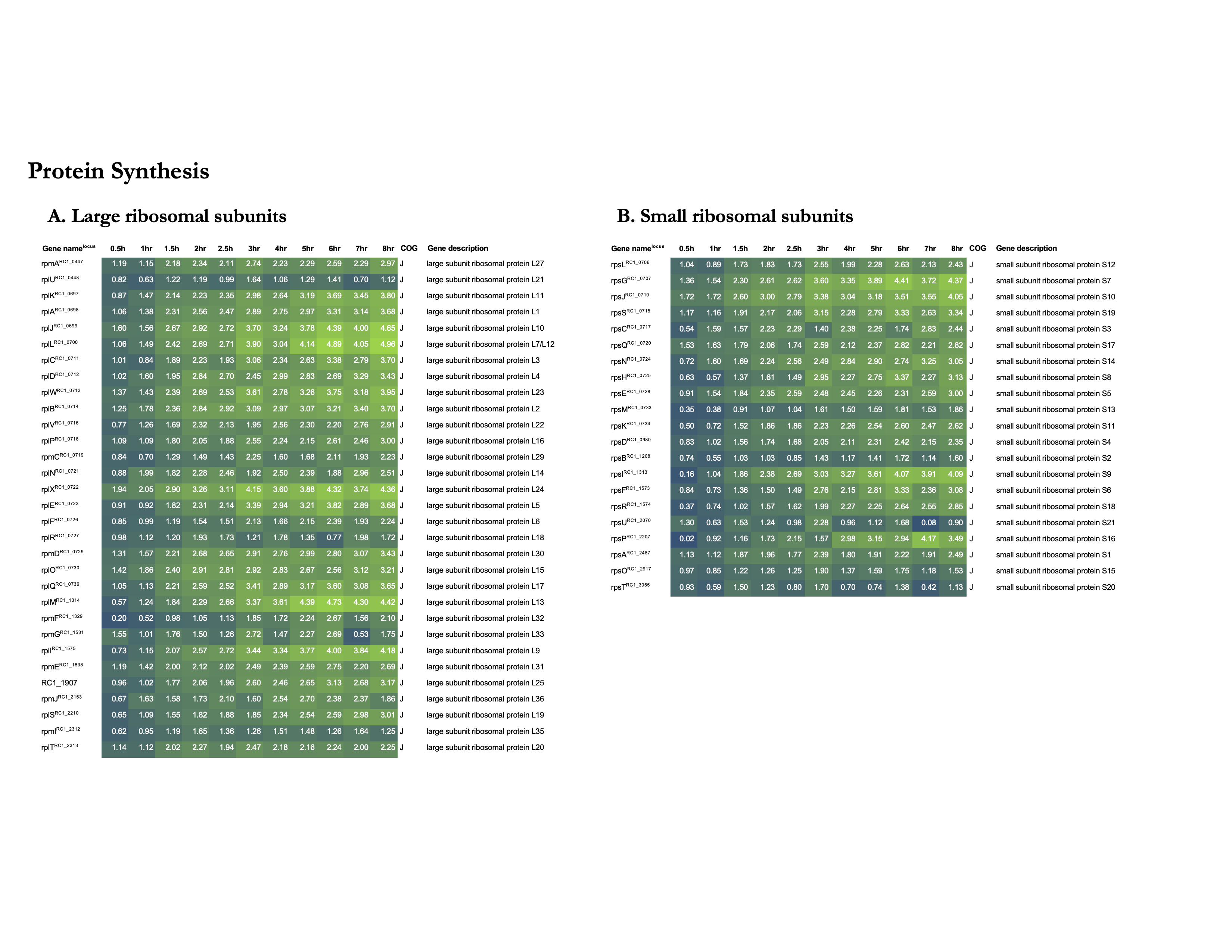

Supplement: S5 Fig — A) and B) show increase expression of genes for large and small ribosomal subunits early in germination, respectively. C) shows changes in expression of ribosomal initiation, elongation and release factors. D) shows expression changes of genes involved in protein folding. E) shows early expression of ftsY, coding for a component of the signal recognition system that targets integral membrane proteins to the membrane. Color of boxes areas noted in S3 Fig and the numbers representing a log2 fold change. (TIFF) [file pgen.1008660.s007.tiff]

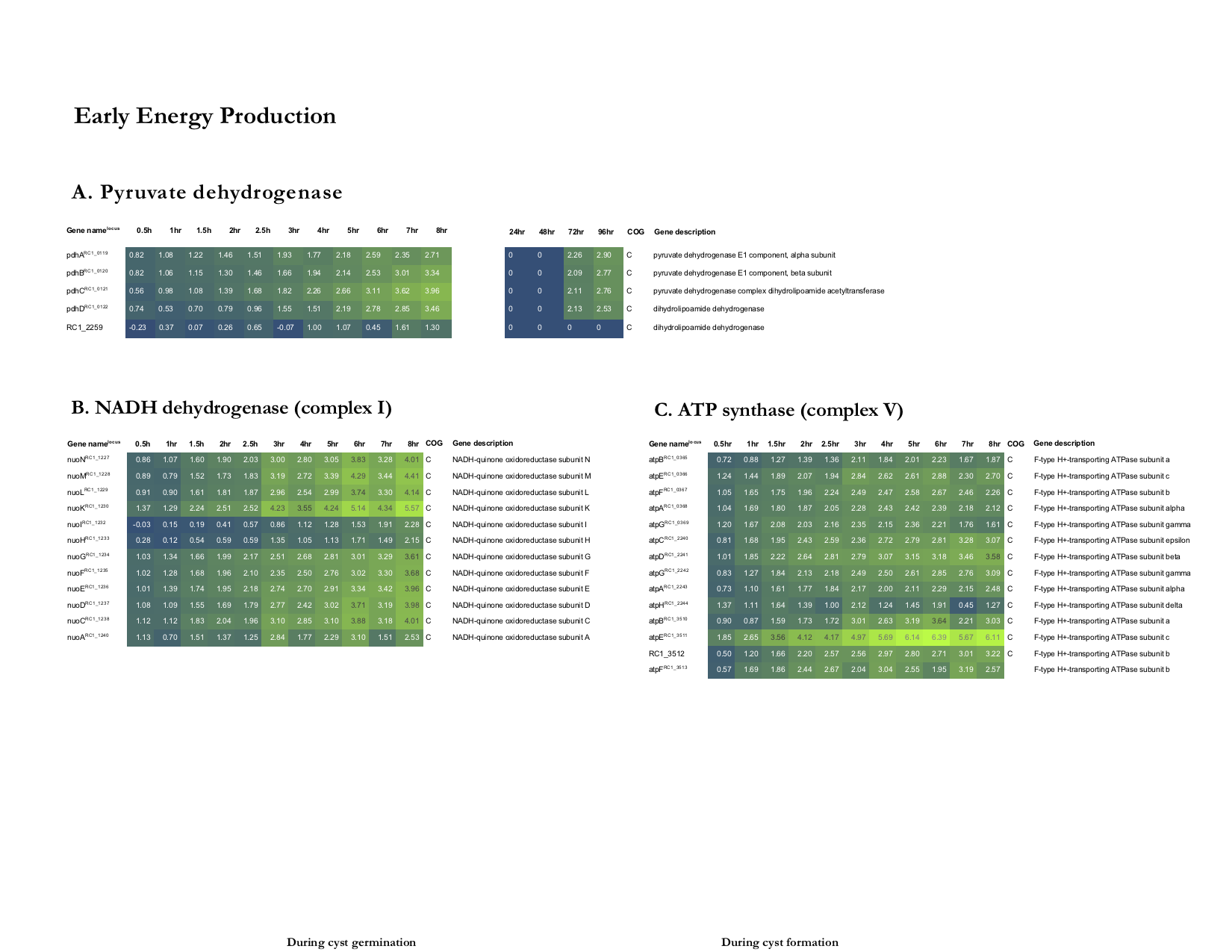

Supplement: S6 Fig — A) early expression of pyruvate dehydrogenase that generates NADH as a product. B) early expression of NADH dehydrogenase (complex I) that utilizes NADH to form a membrane potential and (C) early expression of ATP synthase (complex V) that utilizes a membrane potential for ATP production. Color of boxes are as noted in S3 Fig and the numbers represent the log2 fold change. (TIFF) [file pgen.1008660.s008.tiff]

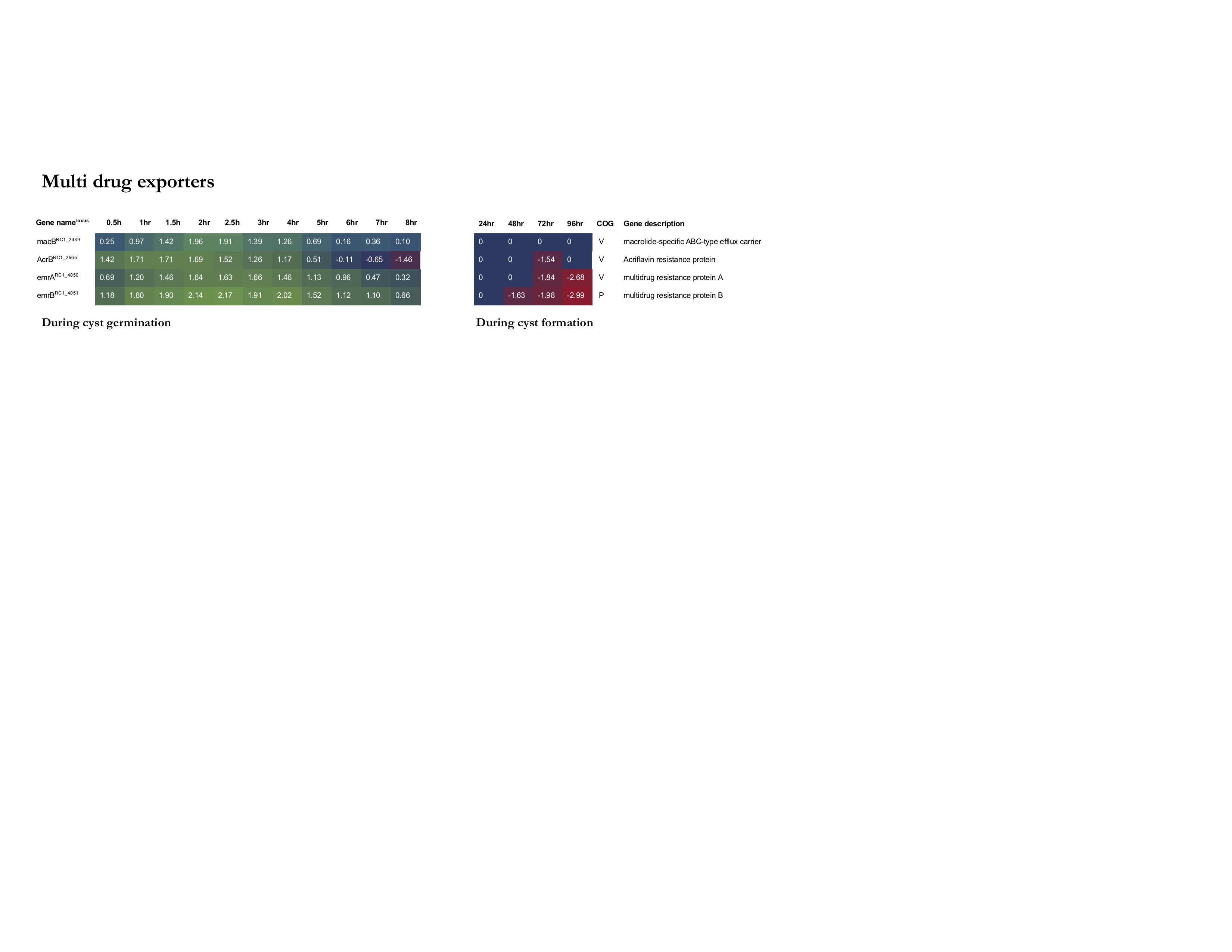

Supplement: S7 Fig — There is also opposing regulation during cyst germination (left heat map) versus cyst formation (right heat map) for several drug export genes. Color of boxes are as noted in S3 Fig and the numbers represent the log2 fold change. (TIFF) [file pgen.1008660.s009.tiff]

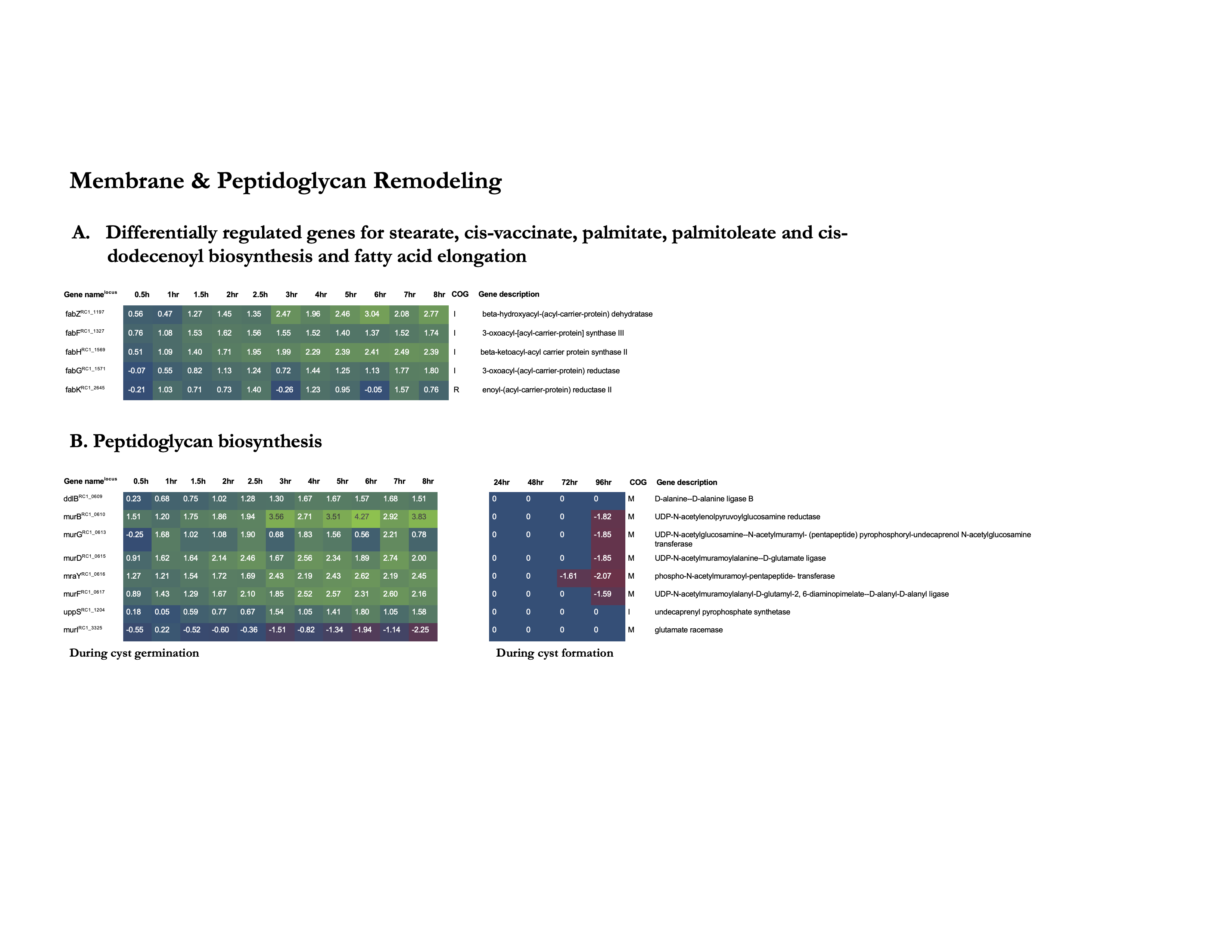

Supplement: S8 Fig — Heat map of gene expression changes that affect fatty acid (A) and peptidoglycan (B) biosynthesis. Color of boxes are as noted in S3 Fig and the numbers represent the log2 fold change. (TIFF) [file pgen.1008660.s010.tiff]

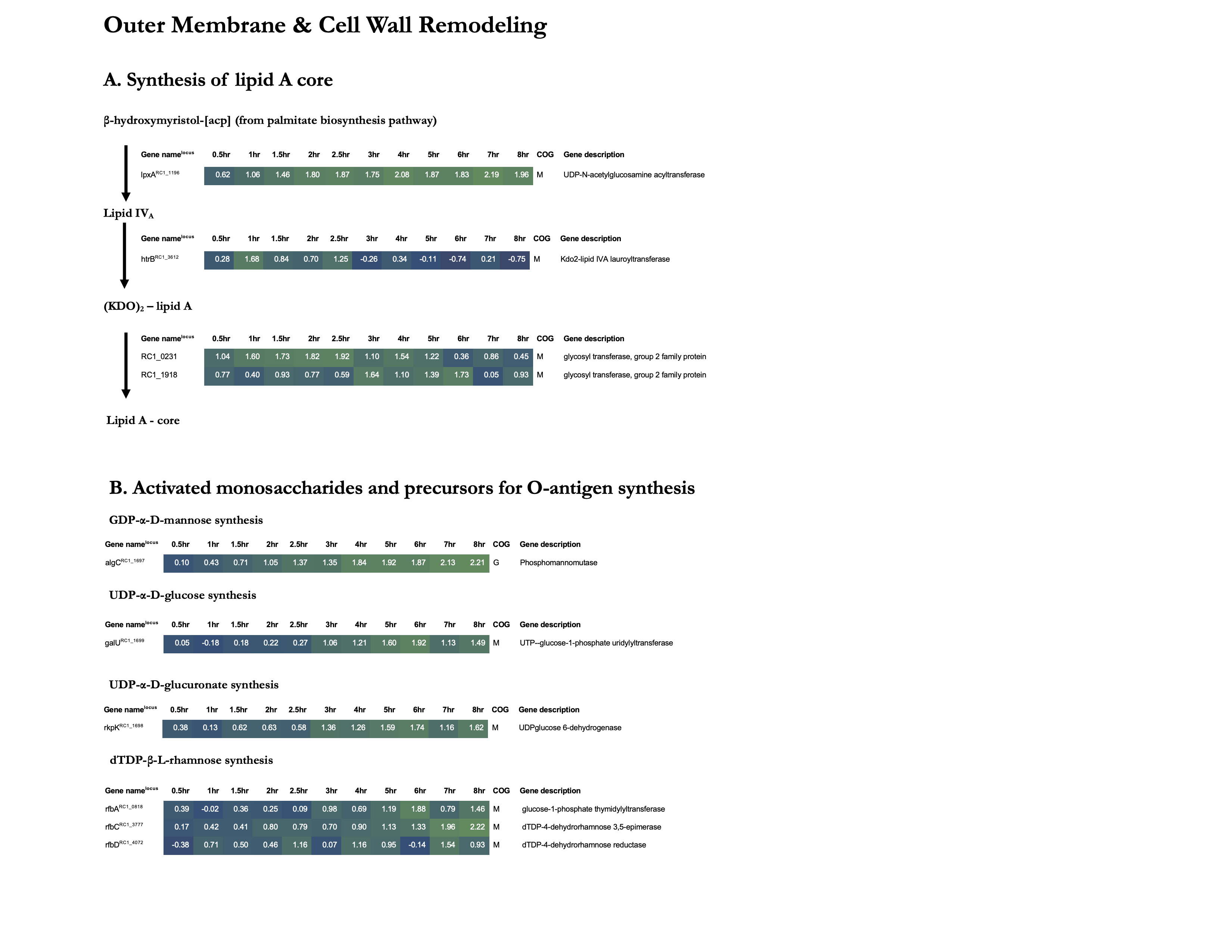

Supplement: S9 Fig — Numerous genes involved in biosynthesis of the lipopolysaccharide lipid A core (A) and the O-antigen (B) that undergo a ramp up in expression. This is contrasted by decreased expression of genes involved in cell wall recycling (C) and genes involved in synthesis of exopolysaccharide precursors involved in cyst cell wall exine synthesis (D). Color of boxes are as noted in S3 Fig and the numbers represent the log2fold change. (TIFF) [file pgen.1008660.s011.tiff]

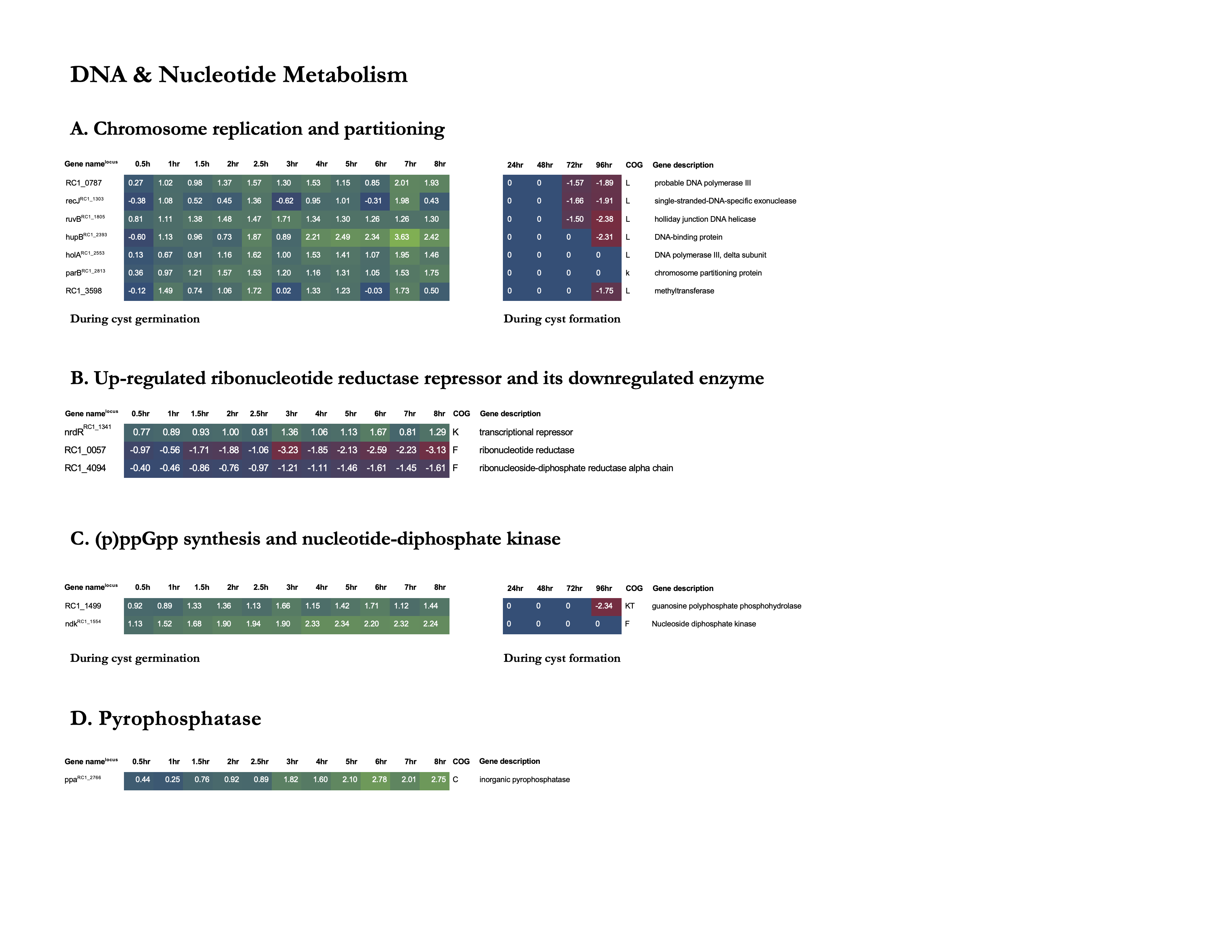

Supplement: S10 Fig — Heat map of genes involved in chromosome replication and partitioning (A) and coding for enzymes involved in nucleotide metabolism (B & C) and pyrophosphatase (D). Color of boxes are as noted in S3 Fig and the numbers represent the log2 fold change. (TIFF) [file pgen.1008660.s012.tiff]

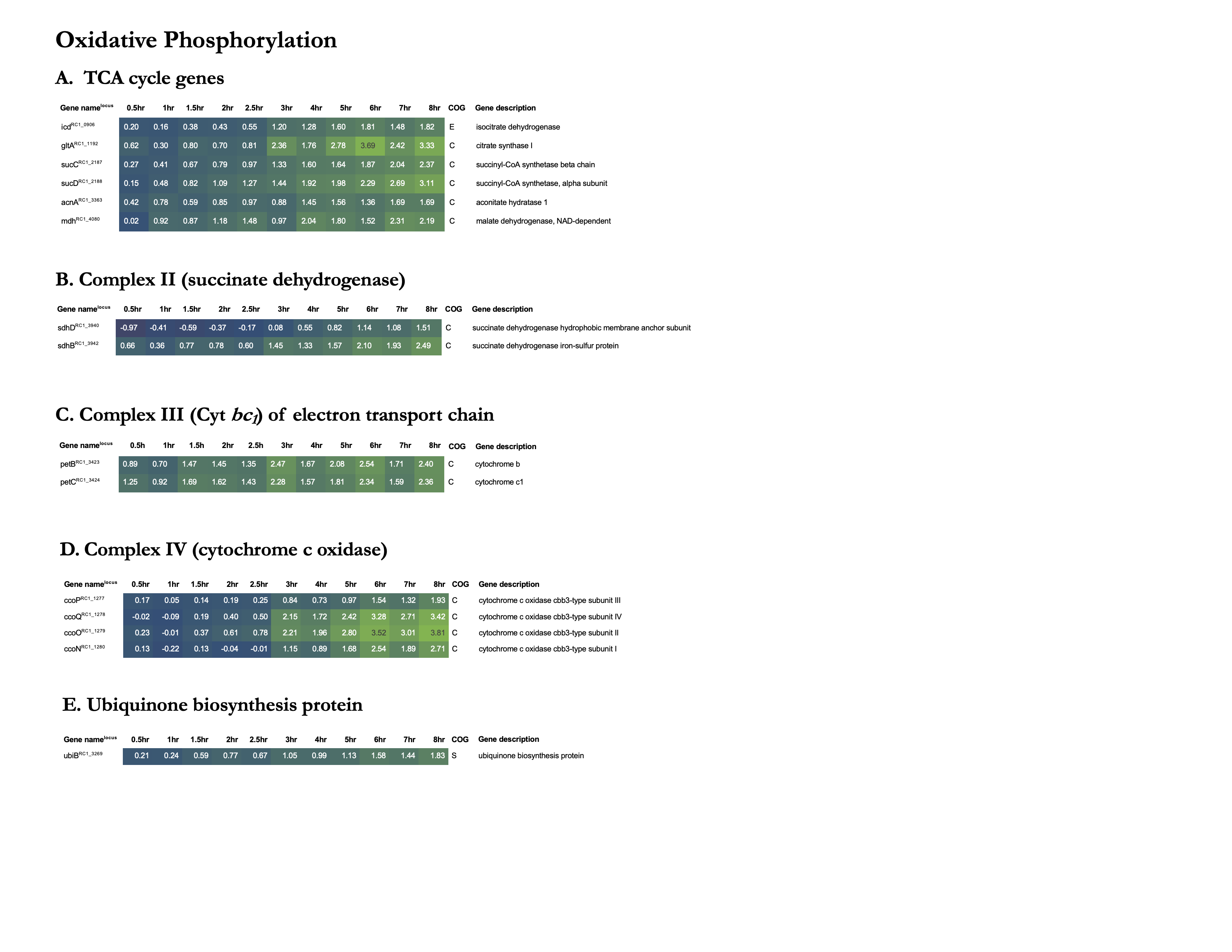

Supplement: S11 Fig — Many genes coding for enzymes in the TCA cycle (A and B) exhibit increased expression midway during germination. Components of the respiratory electron transport chain (C, D and E) also undergo increased expression during a similar period of germination. Color of boxes are as noted in S3 Fig and the numbers represent the log2 fold change. (TIFF) [file pgen.1008660.s013.tiff]

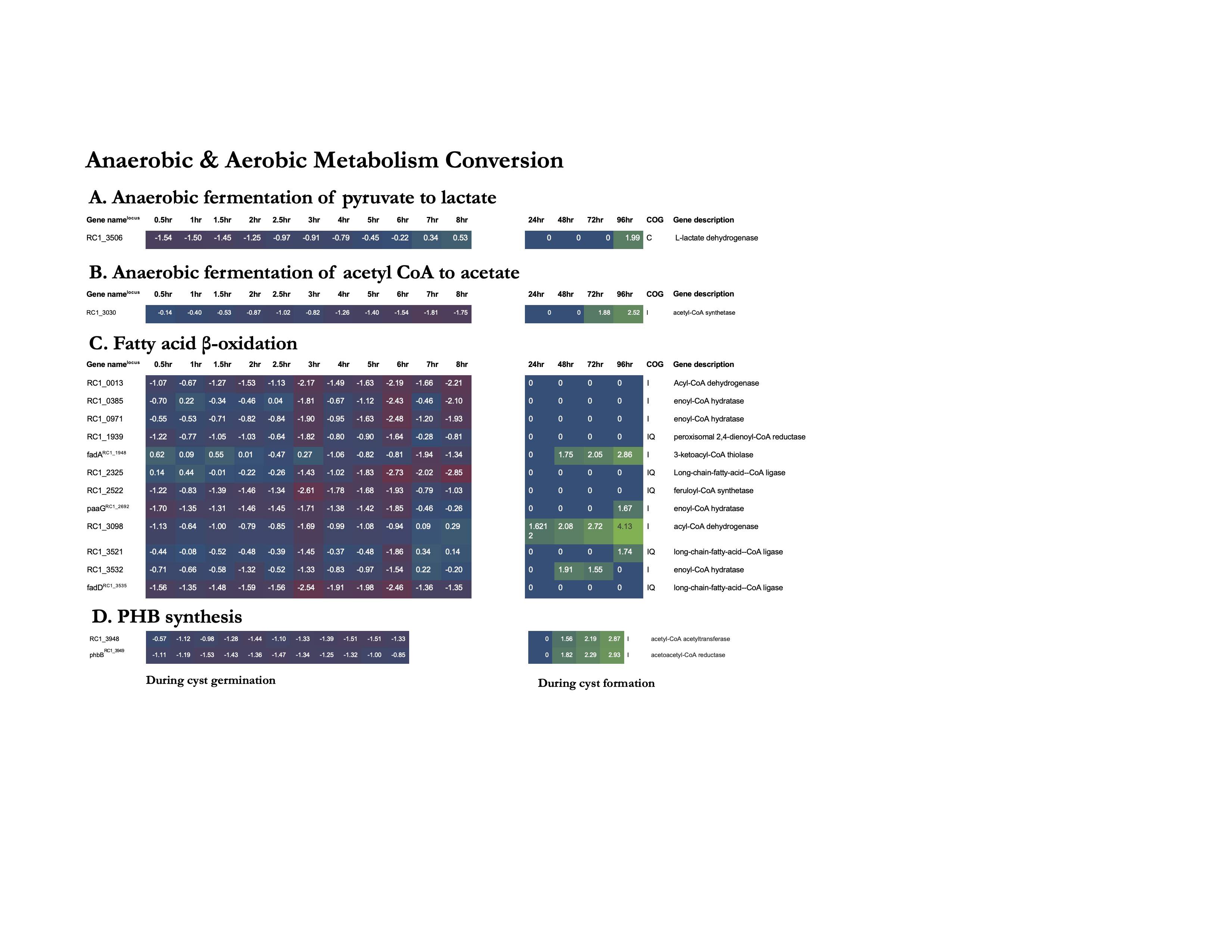

Supplement: S12 Fig — (A & B), reduction in expression of lactate dehydrogenase and acetyl-CoA synthetase that are involved in production of lactate and acetate, respectively. (C) reduced expression of numerous enzymes involved in fatty acid β-oxidation. (D) Reduced expression of enzymes involved in polyhydroxybutyrate (PHB) synthesis during cyst germination. The left heat maps are from RNA-seq data obtained during cyst germination while the right heat maps are derived from independent RNA-seq data during cyst formation. Each of these processes are undergo inverse expression patterns during these different stages of cyst development. Color of boxes are as noted in S3 Fig and the numbers represent the log2 fold change. (TIFF) [file pgen.1008660.s014.tiff]

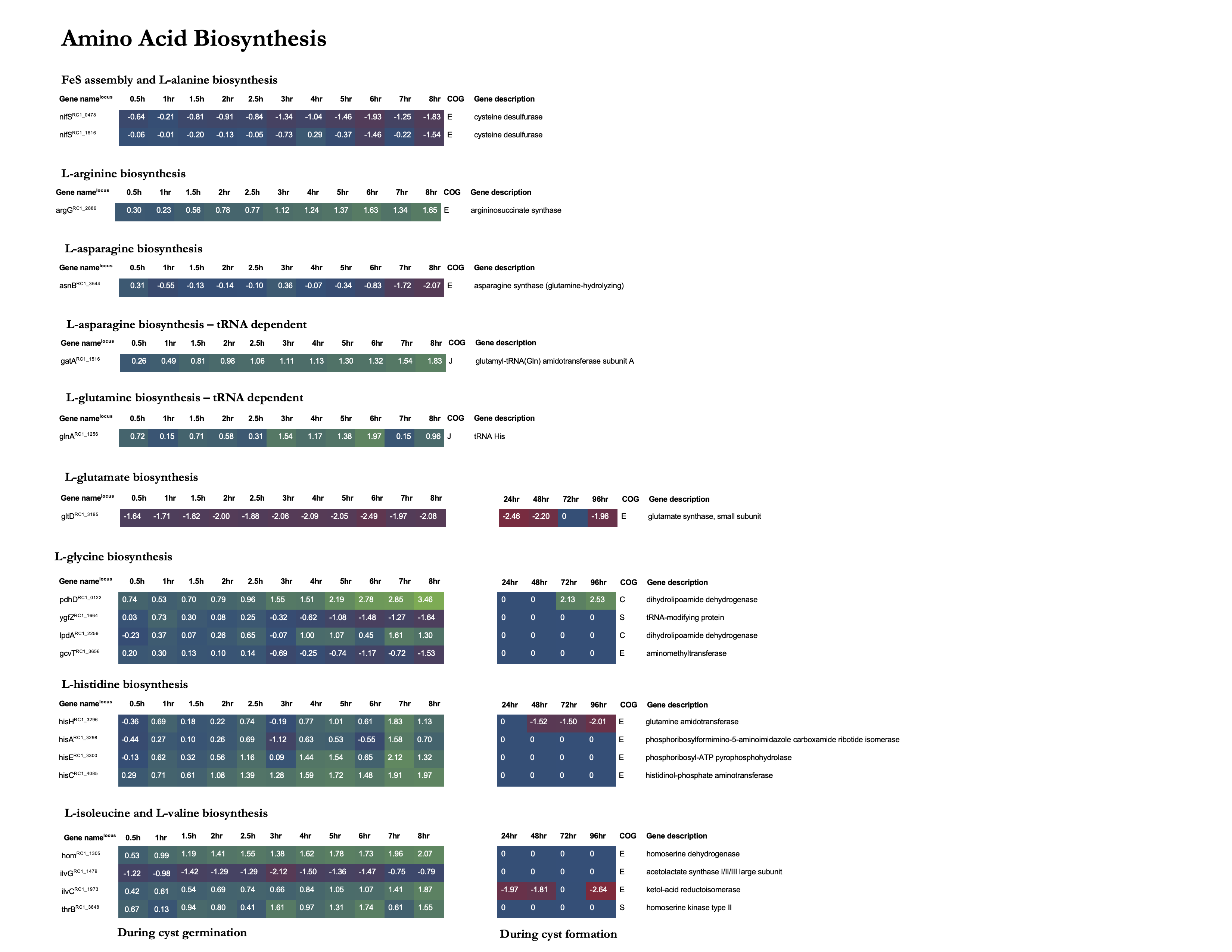

Supplement: S13 Fig — In some cases, there are also expression changes observed during cyst development is shown on the right most heat maps. Color of boxes are as noted in S3 Fig and the numbers represent the log2 fold change. (TIFF) [file pgen.1008660.s015.tiff]

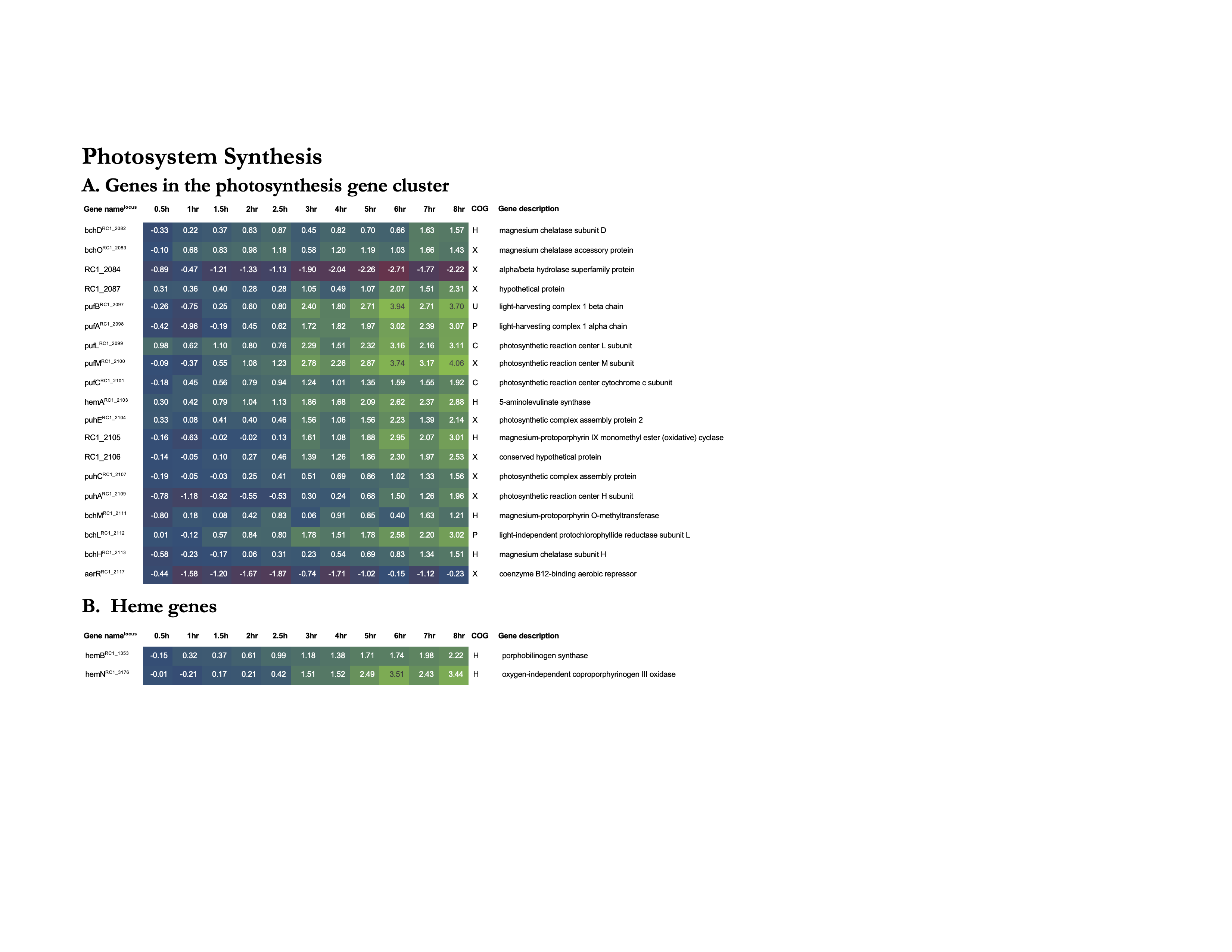

Supplement: S14 Fig — Heat map of genes involved in synthesis of the bacteriochlorophyll, light harvesting and reaction center components of the photosystem (A) as well as several genes involved in the heme and bacteriochlorophyll common trunk of the tetrapyrrole pathway (B). Color of boxes are as noted in S3 Fig and the numbers represent the log2 fold change. (TIFF) [file pgen.1008660.s016.tiff]

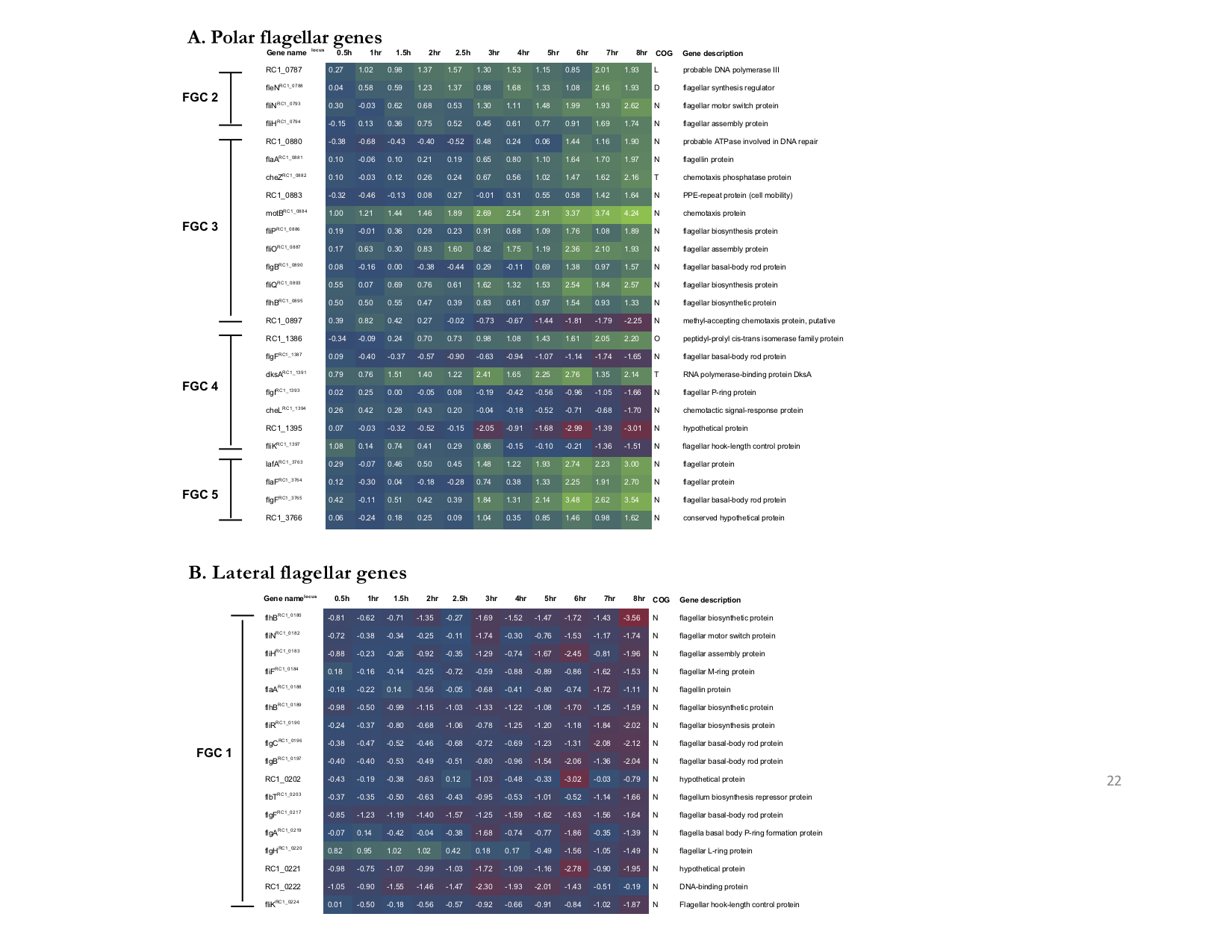

Supplement: S15 Fig — Heat map showing expression profiles of polar (A) and lateral (B) flagellar genes. Operons coding for flagellar gene clusters (FGC) are indicated to the left of the heat maps. (C) is a heat map of the Che2 gene cluster that is involved in the synthesis of lateral flagella and (D) is a heat map of the Che1 gene cluster that is involved in chemotaxis and phototaxis. Color of boxes are as noted in S3 Fig and the numbers represent the log2 fold change. (TIFF) [file pgen.1008660.s017.tiff]

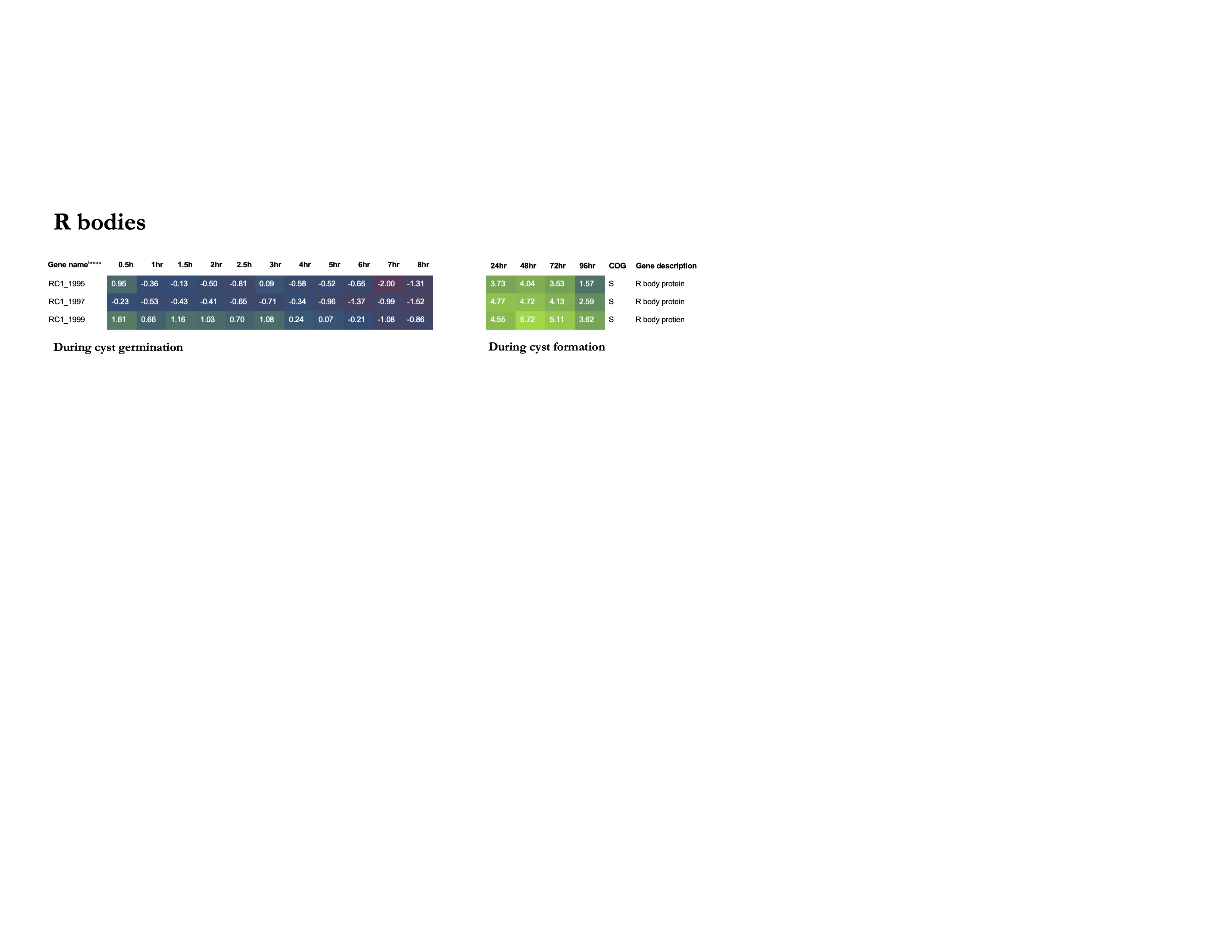

Supplement: S16 Fig — Heat map showing expression profiles of R body genes that goes down during germination (left) and up during cyst formation (right). Color of boxes are as noted in S3 Fig and the numbers represent the log2 fold change. (TIFF) [file pgen.1008660.s018.tiff]

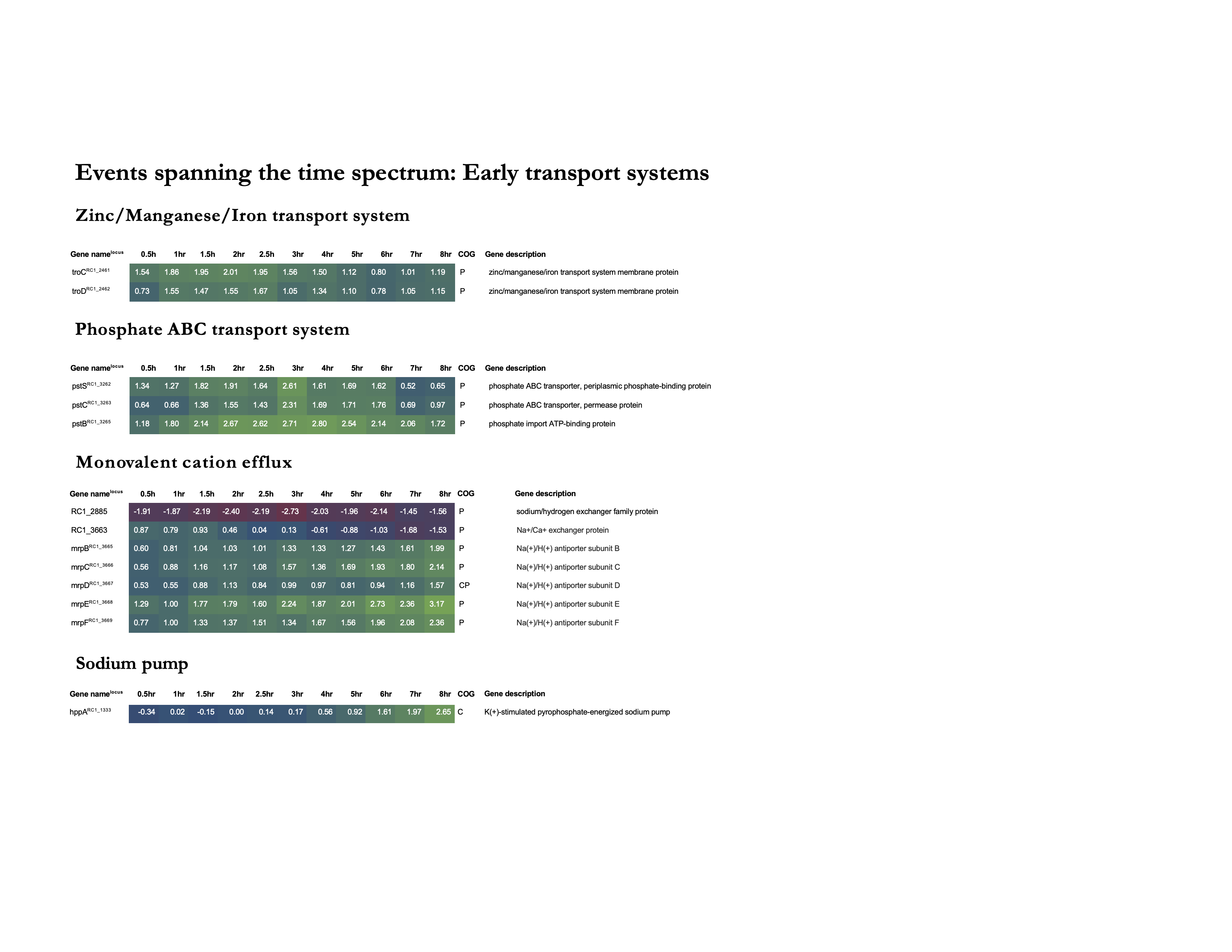

Supplement: S17 Fig — (A) Zinc/manganese/iron transport systems. (B) ABC transporters of phosphate. (C) Efflux transporters for monovalent cations. (D) K+ stimulated pyrophosphate energized sodium pump. Color of boxes are as noted in S3 Fig and the numbers represent the log2 fold change. (TIFF) [file pgen.1008660.s019.tiff]

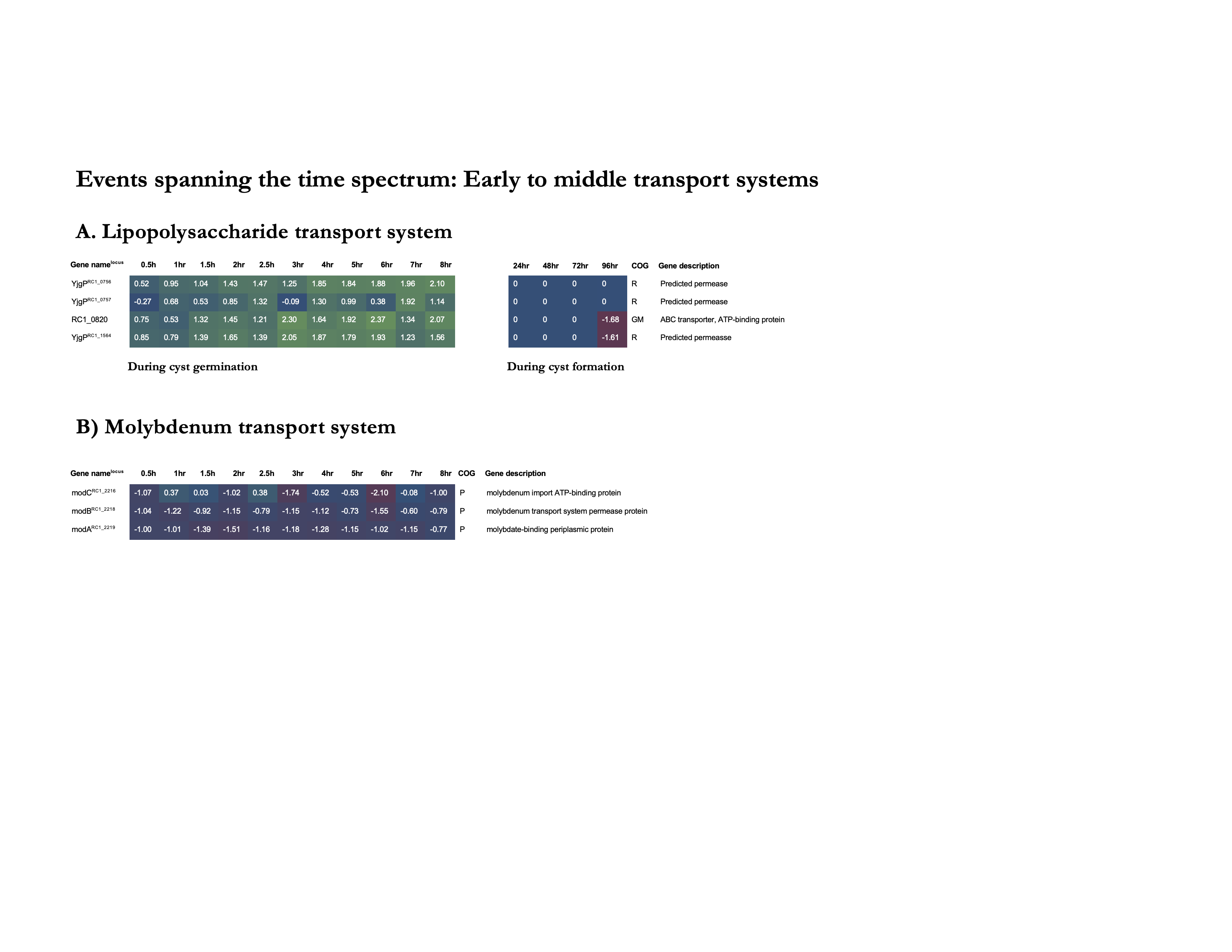

Supplement: S18 Fig — Heatmaps show the expression profiles of the lipopolysaccharide (A) and molybdenum (B) transport systems that are expressed at the early to middle time points of germination. Lipopolysaccharide expression is induced during cyst germination (left heat maps) and reduced during cyst formation (right heat maps). Color of boxes are as noted in S3 Fig and the numbers represent the log2 fold change. (TIFF) [file pgen.1008660.s020.tiff]

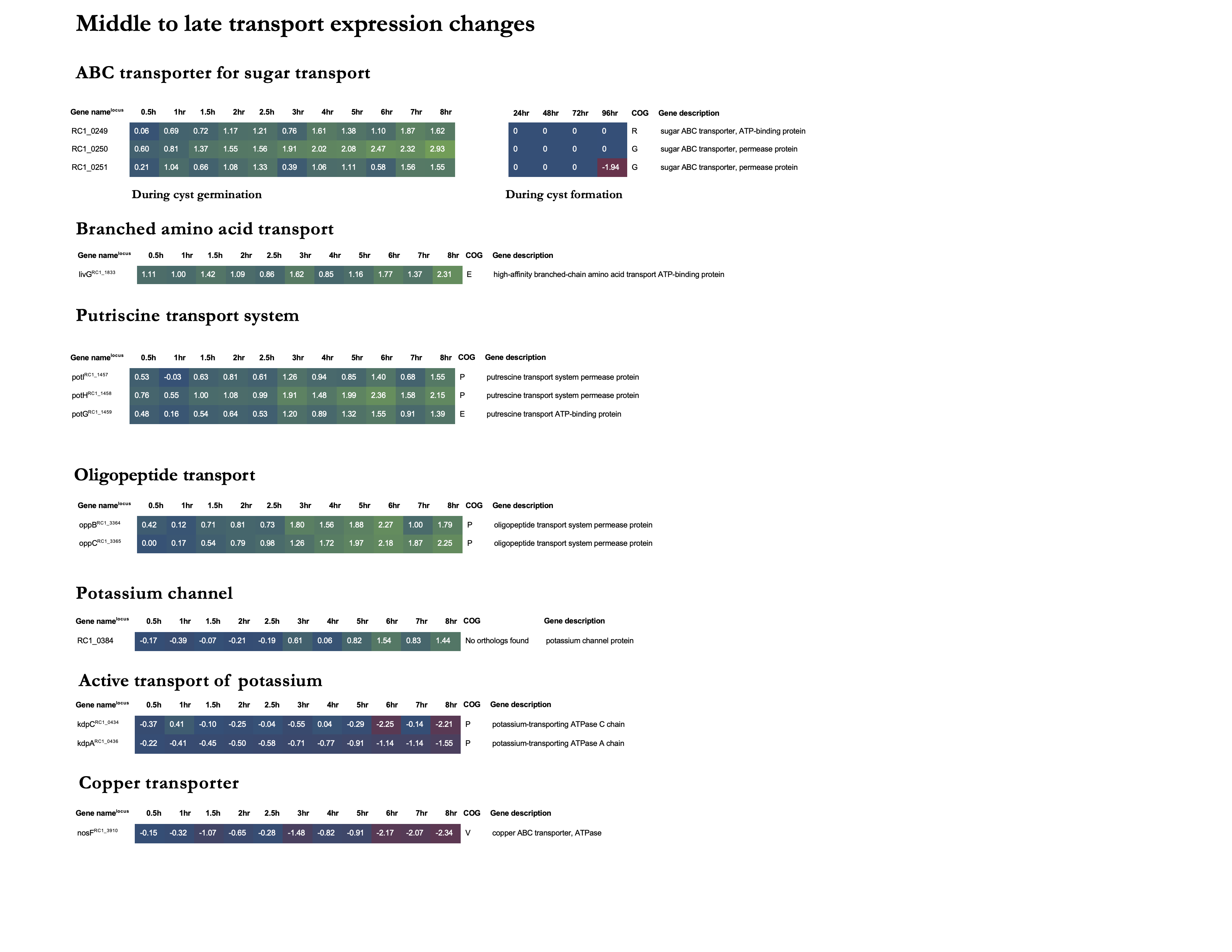

Supplement: S19 Fig — (A) ABC transporters specific for sugar transport. (B) Branched-chain amino acid transport. (C) Putrescine transport. (D) Oligopeptide transport. (E) Potassium transport. (F) Active transport of potassium. (G) Copper transport. Color of boxes are as noted in S3 Fig and the numbers represent the log2 fold change. (TIFF) [file pgen.1008660.s021.tiff]

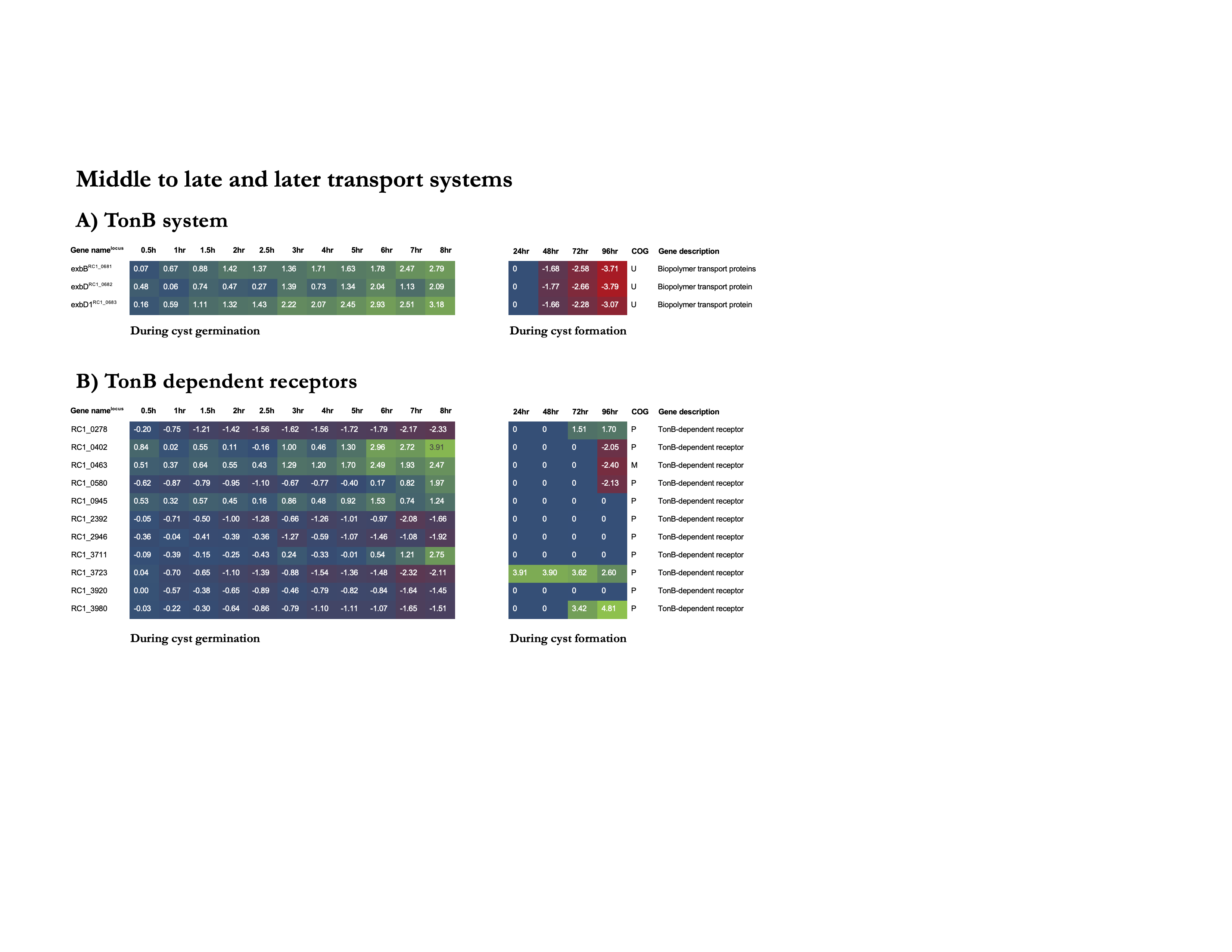

Supplement: S20 Fig — Heatmaps show the middle to late expression profiles of exbB and exbD that code for TonB receptor components involved in transport across the inner membrane (A) and various TonB dependent outer membrane receptors (B). Color of boxes are as noted in S3 Fig and the numbers represent the log2 fold change. (TIFF) [file pgen.1008660.s022.tiff]

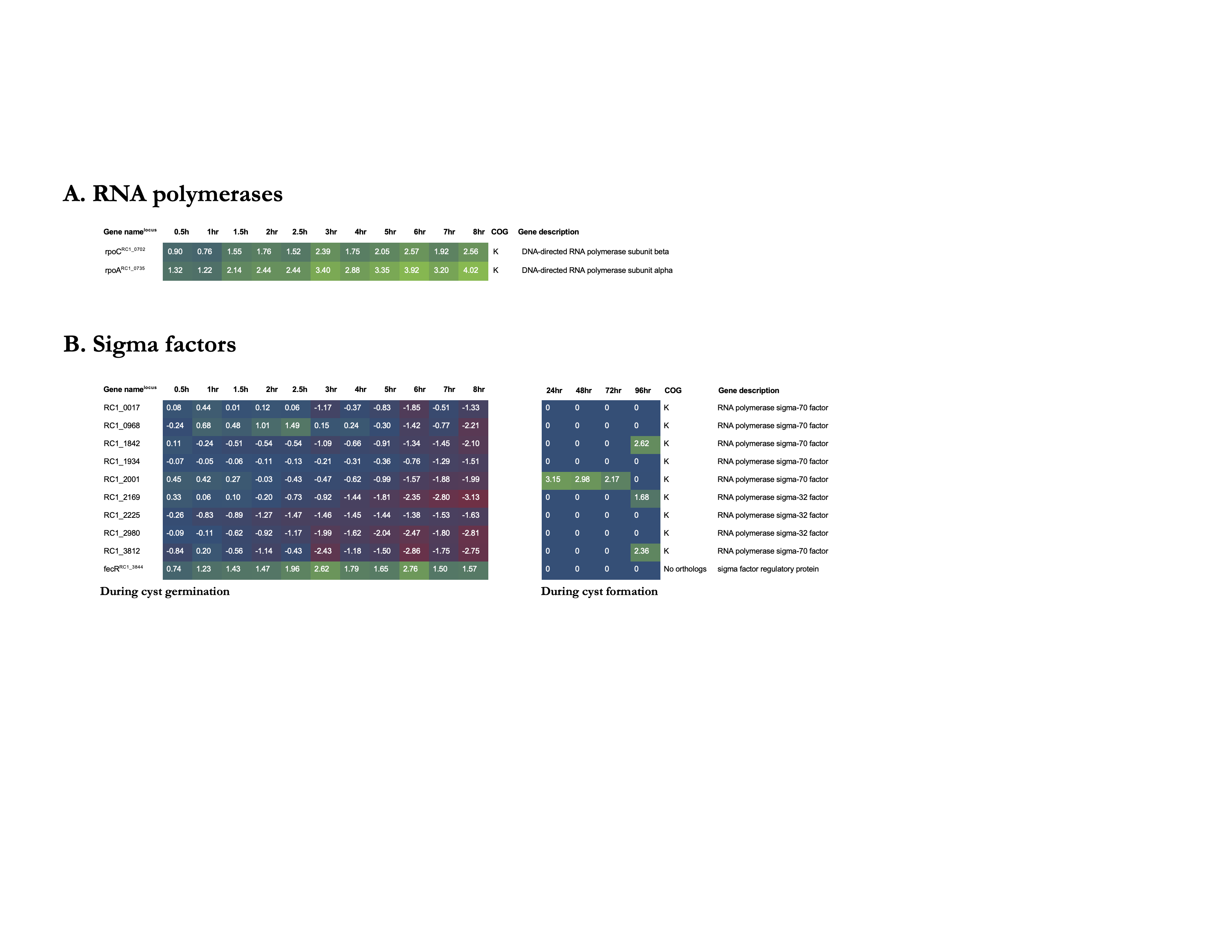

Supplement: S21 Fig — Heatmaps show the expression profiles of holo-enzyme RNA polymerase subunits (A) and sigma factors (B). Color of boxes are as noted in S3 Fig and the numbers represent the log2 fold change. (TIFF) [file pgen.1008660.s023.tiff]

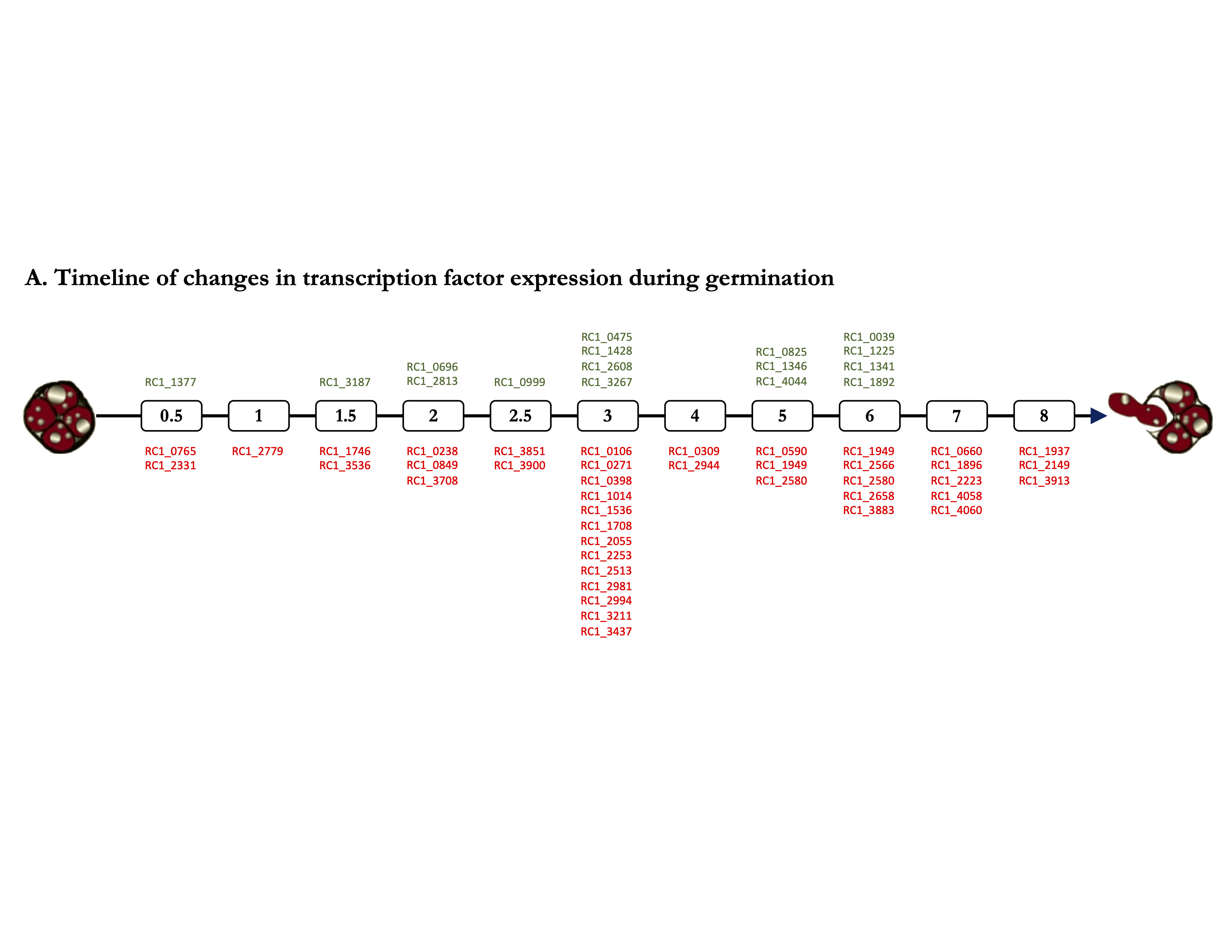

Supplement: S22 Fig — Time line showing the point of significant expression changes of every differentially regulated transcription factor in this dataset (A). Heatmaps showing the expression profiles of down regulated (B) and upregulated (C) transcription factors. Heat maps on the left side of B and D are derived from RNA-seq data sets during cyst germination while the heat maps on the right side are from RNA-seq data sets during cyst formation. Color of boxes are as noted in S3 Fig and the numbers represent the log2 fold change. (TIFF) [file pgen.1008660.s024.tiff]

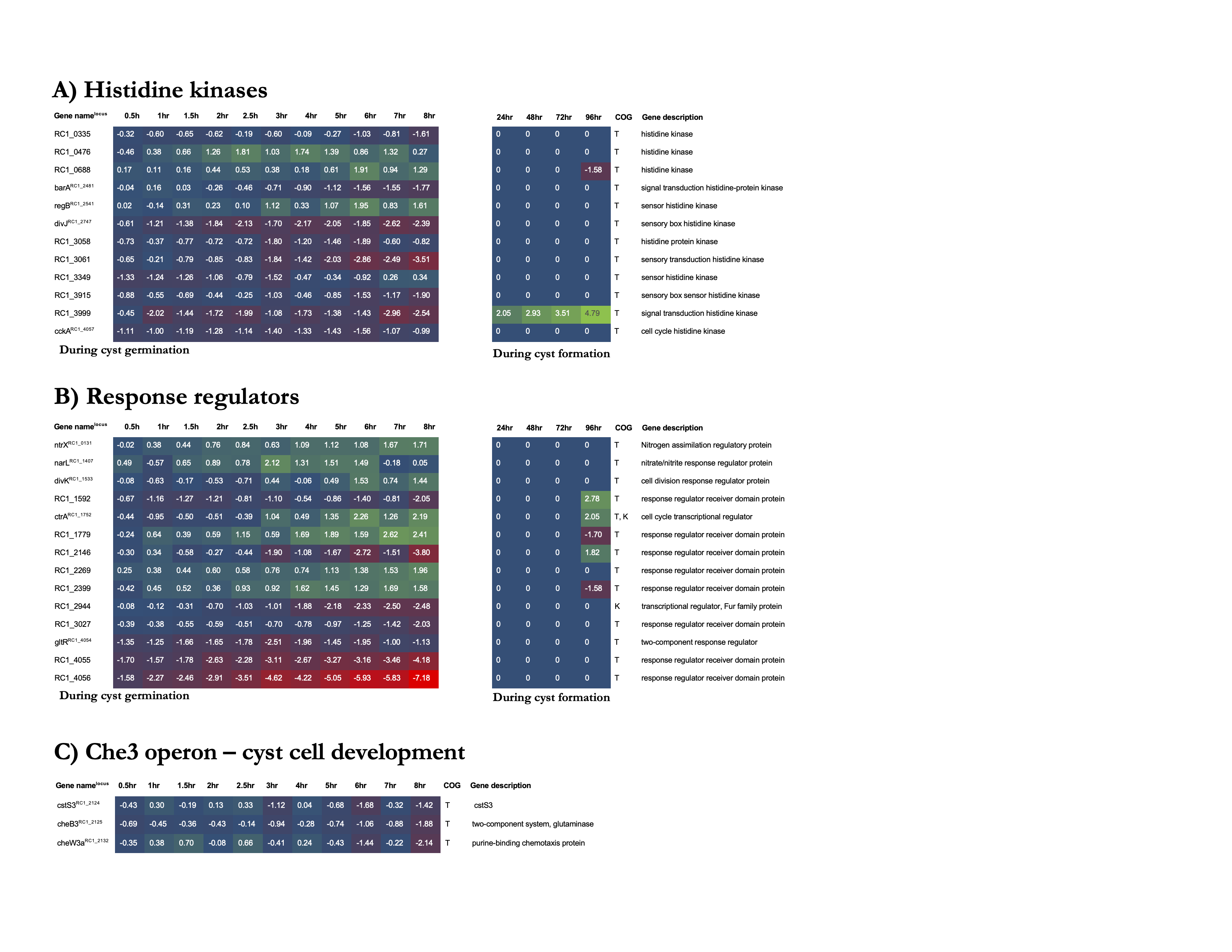

Supplement: S23 Fig — Heatmaps show the expression profiles of histidine kinases (A), response regulators (B) and the che3 gene cluster involved in cyst development (C). Heat maps on the left side are derived from RNA-seq data sets during cyst germination while the heat maps on the right side are from RNA-seq data sets during cyst formation. Color of boxes are as noted in S3 Fig and the numbers represent the log2 fold change. (TIFF) [file pgen.1008660.s025.tiff]
